# Human papilloma virus integration sites and genomic signatures in head and neck squamous cell carcinoma

**DOI:** 10.1002/1878-0261.13219

**Published:** 2022-05-10

**Authors:** Juliette Mainguené, Sophie Vacher, Maud Kamal, Abderaouf Hamza, Julien Masliah‐Planchon, Sylvain Baulande, Sabrina Ibadioune, Edith Borcoman, Wulfran Cacheux, Valentin Calugaru, Laura Courtois, Carole Crozes, Marc Deloger, Elodie Girard, Jean‐Pierre Delord, Antoine Dubray‐Vautrin, Linda Larbi Chérif, Celia Dupain, Emmanuelle Jeannot, Jerzy Klijanienko, Sonia Lameiras, Charlotte Lecerf, Anouchka Modesto, Alain Nicolas, Roman Rouzier, Esma Saada‐Bouzid, Pierre Saintigny, Anne Sudaka, Nicolas Servant, Christophe Le Tourneau, Ivan Bièche

**Affiliations:** ^1^ Department of Genetics Institut Curie PSL Research University Paris France; ^2^ 55216 Department of Drug Development and Innovation (D3i) Institut Curie Paris France; ^3^ Institut Curie Genomics of Excellence (ICGex) Platform PSL Research University Paris France; ^4^ 55216 Department of Medical Oncology Institut Curie Paris France; ^5^ Department of Medical Oncology Hôpital Privé Pays de Savoie Annemasse France; ^6^ Department of Radiotherapy Institut Curie PSL Research University Paris France; ^7^ 56126 Department of Biopathology Centre Léon Bérard Lyon France; ^8^ INSERM U900 Bioinformatics and Computational Systems Biology of Cancer PSL Research University Mines Paris Tech Paris France; ^9^ Department of Medical Oncology and Clinical Research IUCT‐Oncopole Toulouse France; ^10^ Department of Head and Neck Surgery Institut Curie PSL Research University Paris France; ^11^ Department of Pathology Institut Curie PSL Research University Paris France; ^12^ Radiation Oncology Department IUCT‐Oncopole Toulouse France; ^13^ CNRS UMR3244 Institut Curie PSL Research University Paris France; ^14^ 55216 Department of Surgery Institut Curie Saint‐Cloud France; ^15^ Paris‐Saclay University France; ^16^ Department of Medical Oncology Centre Antoine Lacassagne Nice France; ^17^ INSERM 1052 CNRS 5286 Centre Léon Bérard Cancer Research Center Univ Lyon Claude Bernard Lyon 1 University Lyon France; ^18^ 56126 Department of Medical Oncology Centre Léon Bérard Lyon France; ^19^ Pathology unit et Biological Resource Center (BB‐0033‐00098) Centre Antoine Lacassagne Nice France; ^20^ 55216 INSERM U900 Research Unit Institut Curie Saint‐Cloud France; ^21^ INSERM U1016 Faculty of Pharmaceutical and Biological Sciences Paris Descartes University Paris France

**Keywords:** carcinogenesis, head and neck squamous cell carcinoma, HPV copy number, HPV integration, *MYC*, *PDL1*

## Abstract

A prevalence of around 26% of human papillomavirus (HPV) in head and neck squamous cell carcinoma (HNSCC) has been previously reported. HPV induced oncogenesis mainly involving E6 and E7 viral oncoproteins. In some cases, HPV viral DNA has been detected to integrate with the host genome and possibly contributes to carcinogenesis by affecting the gene expression. We retrospectively assessed HPV integration sites and signatures in 80 HPV positive patients with HNSCC, by using a double capture‐HPV method followed by next‐generation Sequencing. We detected HPV16 in 90% of the analyzed cohort and confirmed five previously described mechanistic signatures of HPV integration [episomal (EPI), integrated in a truncated form revealing two HPV‐chromosomal junctions colinear (2J‐COL) or nonlinear (2J‐NL), multiple hybrid junctions clustering in a single chromosomal region (MJ‐CL) or scattered over different chromosomal regions (MJ‐SC) of the human genome]. Our results suggested that HPV remained episomal in 38.8% of the cases or was integrated/mixed in the remaining 61.2% of patients with HNSCC. We showed a lack of association of HPV genomic signatures to tumour and patient characteristics, as well as patient survival. Similar to other HPV associated cancers, low HPV copy number was associated with worse prognosis. We identified 267 HPV‐human junctions scattered on most chromosomes. Remarkably, we observed four recurrent integration regions: *PDL1/PDL2*/*PLGRKT* (8.2%), *MYC*/*PVT1* (6.1%), *MACROD2* (4.1%) and *KLF5/KLF12* regions (4.1%). We detected the overexpression of *PDL1* and *MYC* upon integration by gene expression analysis. In conclusion, we identified recurrent targeting of several cancer genes such as *PDL1* and *MYC* upon HPV integration, suggesting a role of altered gene expression by HPV integration during HNSCC carcinogenesis.

## Graphical abstract

Five previously described mechanistic signatures of human papillomavirus (HPV) integration [episomal, integrated in a truncated form revealing two HPV‐chromosomal junctions colinear (2J‐COL) or nonlinear (2J‐NL), multiple hybrid junctions clustering in a single chromosomal region (MJ‐CL) or scattered over different chromosomal regions (MJ‐SC) of the human genome] are confirmed in our head and neck squamous cell carcinoma cohort. Two hundred sixty‐seven HPV‐human junctions scattered on most chromosomes are reported.

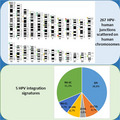

**Abbreviations**
2J‐COL: two colinear junctions
2J‐NL: two nonlinear junctions
2J‐UN: unclassified two junctions
ASCC: anal squamous cell carcinoma
CC: cervical cancer
DFS: disease free survival
ecDNA: hybrid human–virus extra chromosomal DNA
EPI: episomal
HNSCC: head and neck squamous cell carcinoma
HPV: human papillomavirus
KLF: Krüppel‐like factors
M2: pro‐tumoural polarized M2 macrophages
MJ‐CL: multiple hybrid junctions clustered in a single chromosomal region
MJ‐SC: multiple hybrid junctions scattered over different chromosomes
nf‐VIF: nextflow‐based Virus Insertion Finder
NGS: next generation sequencing
OPSCC: oropharynx squamous cell carcinoma
OS: overall Survival
R1: cancer cells present microscopically on surgically resected specimen
SCC: squamous cell carcinoma
TNM: international staging system tumour‐node‐metastasis

## 1 Introduction

More than 90% of head and neck cancers are squamous cell carcinomas (HNSCC), predominantly affecting the oropharynx, but also the oral cavity, hypopharynx and larynx. HNSCC is the seventh most common cancer worldwide [1]. Treatment options for non‐metastatic HNSCC include surgery or radiation therapy (+/− induction or concomitant chemotherapy) [2]. Relapse is frequent: 10–20% of cases for stage I‐II and up to 50% of cases for stage III. Recurrent/metastatic HNSCC are associated with poor prognosis with an overall survival (OS) inferior to one year. New standards of care for recurrent and/or metastatic HNSCC now include PD1 targeting therapies [3, 4, 5].

HNSCC main risk factors are alcohol and tobacco use and human papillomavirus (HPV) infection. The overall HPV prevalence in HNSCC is 26% and reaches up to 80–90% for oropharyngeal squamous cell carcinomas (OSCC) [6]. A recent review highlighted that the incidence of HPV‐related HNSCC cancers increased in the last decades regardless of age group [7]. HPV role in tumour initiation and progression is clearly established in cervical, anal canal, oropharynx, vulva and penile carcinomas [8].

HPV is a small double‐strand DNA virus of approximately 8000 base pairs. More than 200 genotypes have been identified, among which 13 high risk HPVs are recognized as carcinogens by the International Agency for Research on Cancer. During the oncogenic process, the viral genome can be maintained as extrachromosomal nuclear episomes and/or it can be integrated into the host genome [9]. Carcinogenesis is primarily driven by *E6* and *E7* viral oncogenes when HPV remains as an episome or if integration happens outside a gene. Then, E6 and E7 oncoproteins expression respectively disrupts p53 and pRb tumour suppressors. Whereas, integration into or near a human cancer related gene can affect the gene expression and directly contribute to carcinogenesis *via* chromosome instability, gene disruption, or regional amplifications within the cellular genome [10]. HPV integration has been reported to cause E1/E2 disruption and expression of alternate E6 transcripts [10, 11, 12, 13].

We previously developed the HPV double capture method followed by next‐generation sequencing (NGS) to: (a) precisely determine HPV genotypes, (b) characterize the HPV sequence (full‐length when HPV remained episomal or truncated when integrated), (c) analyze the viral‐host DNA junctions, (d) quantify the HPV copy number per sample and (e) assess the sites and signatures of HPV integration into the human genome [14]. We previously identified five mechanistic signatures in the small cohort of cervical cancer. The signatures were: episomal (EPI), integrated in a truncated form revealing two HPV‐chromosomal junctions colinear (2J‐COL) or non‐linear (2J‐NL), multiple hybrid junctions clustering in a single chromosomal region (MJ‐CL) or scattered over different chromosomal regions (MJ‐SC) of the human genome. Our team recently applied this technique in large cohorts of HPV‐positive anal squamous cell carcinomas (ASCC) [15] and cervical cancer (CC) patients [16] to assess prognostic and predictive value of these HPV genomic signatures.

Several studies across different tumour localizations focused on HPV integration and its impact on gene expression and immune microenvironment modulation, but much remains to be confirmed [10, 17].

Nonintegrated HPV has been previously reported to exert oncogenic effects while maintaining status as viral circular DNA in oropharynx squamous cell carcinoma (OPSCC) [21] Viral integrations were also reported to induce human–viral hybrid amplicons containing both viral DNA and segments from the human genome [22], suggesting three categories of HPV DNA including, episomal, integrated and human–viral episomal hybrids [23]. Hybrid human–virus extra chromosomal DNA (ecDNA) was shown to induce an increase in HPV oncogenes E6 and E7 copy number. More recently, the interaction of the HPV genome with cancer‐associated ecDNA was shown to induce high expression of hybrid viral–human oncogene transcripts in OPSCC [24].

Previous reports in the literature identified HPV integration sites in different types of squamous cell carcinomas and particularly in cervical cancer. Frequent sites were reported in the *MYC, TMEM49, FANCC* and *RAD51B* genes. Conversely to cervical cancer, few studies documented in the literature report HPV integration sites in HNSCC. Parfenov et al. revealed some similarities in HNSCC, with HPV integration events targeting *RAD51B*, *NR4A2* and *TP63* [18, 21, 25, 26].

Studies in cervical cancers showed that episomal HPV confers a relatively favorable survival as compared to integrated HPV [27, 28, 29]. In HNSCC, high HPV copy number was correlated with the episomal status associated with a significantly better prognosis. On the other hand, low HPV copy numbers were correlated with HPV integration and a poor prognosis [29, 30]. Another study suggests that an intact E2 gene is correlated to good prognosis [31].

In the current manuscript, we will assess HPV integration sites and signatures and their prognostic value in HNSCC patients. We aimed to identify integration hotspots implicated in oncogenesis. We also evaluated the impact of HPV integration on the expression of major cancer and immune‐related genes.

## 2 Methods

### 2.1 Patients and samples

Eighty‐two HPV positive frozen tumour samples from initial biopsies or surgical specimens from 80 patients were retrospectively retrieved from the tissue bank of four French hospitals: Institut Curie, Paris (BB‐0033‐00048), IUCT‐Oncopole, Toulouse (BB‐0033‐00014), Centre Léon Bérard, Lyon (BB‐0033‐00050), and Centre Antoine Lacassagne, Nice (CRB‐CAL/ BB‐0033‐00098). All patients were treated for localized or locally advanced HPV positive HNSCC between 1997 and 2017. Samples were obtained prior to treatment. Matched initial tumour sample and synchronous node sample from cervical node dissection were available for two patients. According to the French regulations and ethical requirements, patients were informed about the research performed on tissue specimens and did not express opposition. The study methodologies conformed to the standards set by the Declaration of Helsinki. The analyses were approved by the internal review board.

### 2.2 Genomic DNA and total RNA extractions

Tumour samples were frozen in liquid nitrogen in a cryotube immediately after surgery and stored at −80 °C. Tumoural cellularity was verified systematically by performing cryosections and by macro‐dissecting tumoural zones. Total genomic DNA was extracted with phenol‐chloroform after proteinase K digestion, followed by the precipitation of nucleic acids in ethanol [32]. Total RNA was extracted with miRNeasy Mini kit Qiagen following supplier’s recommendations. The quality of RNA was verified by migration on agarose gel. Nucleic acids were quantified using Nanodrop spectrophotometer ND‐1000 (ThermoScientific, Wilmington, DE, USA).

### 2.3 HPV genotyping

HPV detection and genotyping were first performed using real‐time PCR. The techniques and primers have been previously described [33, 34].

### 2.4 DNA library preparation

The DNA libraries were prepared using 500 ng of genomic DNA. The fragmentation used ultrasonication (Covaris) to produce double‐strand DNA fragments of 290 bp average length. The Kapa Hyper Prep kit Roche was used for the next steps, according to the manufacturer’s instructions: (a) End‐Repair and A‐tailing, (b) ligation of adapters, containing unique barcodes for each sample, specific to the Illumina (San Diego, CA, USA) technology for amplification and sequencing, (c) purification and size selection with Agencourt AMPure XP, (d) pre‐capture PCR and (e) purification.

### 2.5 HPV double capture method and NGS sequencing

The double capture was carried out using the SeqCap EZ Rapid Library Small Target Capture method, developed by Roche NimbleGen, which is adapted to capture small DNA targets [14]. The DNA libraries were multiplexed by 12 samples and hybridized with the biotinylated HPV oligonucleotide probes that recognize all HPV subtypes. The DNA sequences were then captured by streptavidin beads and amplified by PCR. We performed two rounds of hybridization and capture to improve the specificity and to enrich the fraction of relevant reads for subsequent bioinformatics analysis. Post‐capture libraries were sequenced using Illumina MiSeq system, in 150 bp paired‐end reads, with 24 samples multiplexed on a V2 micro flow‐cell.

### 2.6 Bioinformatics pipeline for analysis of HPV genotypes and viral‐cellular junctions

To simplify the post‐sequencing analyses, raw sequencing reads were analyzed with the nf‐VIF (nextflow‐based Virus Insertion Finder) bioinformatics pipeline (v1.0.1) [16]. The pipeline is available for free public access: https://github.com/bioinfo‐pf‐curie/nf‐VIF. Briefly, nf‐VIF was developed to detect and genotype the HPV strain(s) in the samples and to precisely map the integration sites on the Human genome. The different steps of the analysis are; (a) reads cleaning and quality controls, (b) HPV genotyping, (c) local alignment on detected HPV strain(s), (d) detection of putative HPV breakpoints using soft‐clipped reads, (e) soft‐clipped reads alignment on Human genome reference, (f) detection of integration loci and filtering of the results and (g) presentation of results in a dynamic report. nf‐VIF reports the number of reads and the positions of the HPV/human junctions with their genomic positions. We used the GRCh37/hg19 on the UCSC Genome Browser (https://genome.ucsc.edu/) to identify the corresponding genes. When the breakpoints were intergenic, we recorded the closest 3’ and 5’ genes.

Additionally, viral integration status was determined using three parameters: (a) human‐virus DNA junction detection as described above, (b) ratio of viral genes E2 and E6 read depth and (c) E2 deleted fraction (i.e., fraction of the gene locus without coverage). Using strain specific coordinates, the depth of coverage was computed from the aligned BAM files using the R–Bioconductor package GenomicAlignments1 (package version 1.28.0, R version 4.1). Samples with no junction detected were considered to exclusively host episomal HPV while samples with at least one junction detected and E2 gene loss (i.e., E2/E6 mean read depth ratio of less than 0.1 or E2 deleted fraction higher than 10%) were classified as hosting fully integrated HPV. Remaining cases were labelled as mixed, having both integrated viral DNA and episomal forms [35] (Tables S1 and S2, Fig S1A and B).

### 2.7 HPV copy number

HPV copy number was calculated as the ratio of the number of reads mapped to HPV genome over the number of reads mapped to the human *KLK3* gene used as a diploid control gene. *KLK3* was chosen as a reference since its size is similar to HPV genome and is not altered in HNSCC. Similar results were obtained with two other reference genes (*GAPDH* and *RAB7A*). To determine the optimal cutoff value for HPV copy number, we used CutoffFinder.R. This tool allows cutoff optimization for biomarkers that are investigated in research [36].

### 2.8 Description of HPV genomic signatures

The absence of HPV‐human chromosomal junction reads indicated that the viral genome was maintained in its episomal form: this signature was named EPI. In the presence of HPV integration, the viral genome and its breakpoints as well as the coordinates of the integration sites in the human genome were identified by pure HPV reads and HPV‐human chromosomal junction reads. Integration in a truncated form revealing two HPV‐chromosomal colinear junctions is called 2J‐COL or non‐linear named 2J‐NL. Integration with multiple hybrid junctions clustering in a single chromosomal region is referred as MJ‐CL or MJ‐SC when junctions are scattered over different chromosomal regions (MJ‐SC) of the human genome. In some cases, we identified a single HPV‐chromosomal junction corresponding to two junctions with a lost junction; it was named 2J‐UN.

### 2.9 Real‐time quantitative reverse transcription PCR

To characterize the consequences of HPV integration on gene expression we performed real‐time quantitative PCR. We designed primers for our integration hotspot genes: *PLGRKT, PDL1* (also known as *CD274), PDL2* (also known as *PDCD1LG2* or *CD273*), *MYC* and *PVT1*. Total RNA was available for 44 patients including three among the four patients with integration in the *PDL1/PDL2/PLGRKT* region, and two among the three patients with integration in the *PVT1/MYC* region. No RNA was available for the patients with integration near *MACROD2* and *KLF5/KLF12* regions. We selected 51 additional genes mainly involved in the immune process, in particular 17 coding for immune checkpoints, 14 for immune cell populations and 8 for chemokines.

cDNA synthesis and PCR‐reaction conditions have been previously described elsewhere [37]. Target gene expression levels were normalized on the basis of *TBP* contents (Genbank accession number NM_003194) used as an endogenous RNA control. The expression values of the tumour samples were normalized such that the median of the expression values of 13 normal head and neck tissues was one.

Primers for target genes were designed with the assistance of Oligo 6.0 computer program (National Biosciences, Plymouth, MN). To avoid amplification of contaminating genomic DNA, one of the two primers was placed at the junction between two exons or on two different exons. Agarose gel electrophoresis was used to verify the specificity of PCR amplicons. The sequences of the oligonucleotide hybridization primers are available upon request.

### 2.10 Copy number variant analysis using 500 gene panel

Sequencing was performed using an in‐house NGS panel of 571 genes, called DRAGON Dx (Detection of Relevant Alterations in Genes involved in Oncogenetics). Indexed paired‐end libraries of tumour DNA were performed using the Agilent Sureselect XT2 library prep kit. The kit supports sequencing targeted regions of the genome spanning 2.7 Mb. About 50 ng of input DNA were used to build the libraries according to the manufacturer’s protocol. The pool was finally sequenced on a NovaSeq 6000 (Illumina) S2 × 150 bp flow cell.

Targeted chromosomal regions were annotated according to UCSC database [38]. For each region, reads were counted and a double normalization was performed: (a) normalization by the total number of reads in each sample and (b) normalization by median coverage of all samples. After normalization, the reference value was 1.

### 2.11 Statistical analysis

To compare signatures’ repartition between HNSCC, ASCC and CC we used Chi‐square test. Correlations between HPV genomic signatures and clinical, histological and molecular features were assessed using a Chi‐square test (with Yates correction if appropriate). Given the small sample size, we pooled the 2J‐UN, 2J‐COL and 2J‐NL together and the MJ‐CL and MJ‐SC together. The three groups were EPI, 2J and MJ. Overall survival (OS) was defined as the time interval from the date of diagnosis to death. Disease free survival (DFS) was defined as the time interval from diagnosis to the date of relapse. Survival data were censored at the date of last follow‐up. We draw the survival curves using the Kaplan–Meier method and compared them using the log‐rank test. The optimal cutoff value for the HPV copy number was established using CutoffFinder.R. A low HPV copy number was <9 and a high HPV copy number was ≥9. To compare gene expression levels according to the signature group, we used non‐parametric tests (Mann–Whitney or Kruskal–Wallis depending on effectives). For all statistical tests, the limit of significance was defined as *P* < 0.05. All analyses were performed using Prism 6.0 software (GraphPad Inc., La Jolla, CA, USA).

## 3 Results

### 3.1 Patient characteristics and association with survival

Eighty patients with HPV positive HNSCC were included. Tumours were squamous cell carcinoma arising from the oropharynx (*n* = 73/80, 91.2%) or other head and neck anatomical sites (*n* = 7/80, 8.8%). Patients’ characteristics, tumour’s characteristics and treatments are presented in Table 1. Eight patients (*n* = 8/79, 10.1%) were treated by surgery (alone or post‐induction chemotherapy), 15 patients (*n* = 15/79, 19%) had exclusive radiation therapy as a local treatment (with or without concomitant chemotherapy) and 54 patients (*n* = 54/79, 68.4%) had surgery and additional radiation therapy indicated for a R1 resection and/or invasion of the nodal capsule. Two patients did not receive any curative treatment because of impaired general status. As expected age (*P* = 0.02) and tumour stage (*P* = 0.002) were associated with OS.

**Table 1:** Clinical and biological characteristics of 80 patients with HPV positive head and neck squamous cell carcinoma, and association with overall survival. OS, overall survival; NS, non‐significant; CT, chemotherapy; RT, radiotherapy.

Heading	Patients (%)	Events (%)	OS (*P*‐value)^h^
Total	80 (100)	26 (32.5)	
Age (years)			
<65	45 (56.2)	10 (22.2)	
≥65	35 (43.8)	16 (45.7)	**0.02**
Gender			
Male	60 (75)	21 (35)	
Female	20 (25)	5 (25)	0.44 (NS)
Tobacco^a^			
Yes	48 (60.8)	18 (37.5)	
No	31 (39.2)	7 (22.6)	0.32 (NS)
Alcohol^b^			
Yes	19 (25)	7 (36.8)	
No	57 (75)	18 (31.6)	0.7 (NS)
Localization			
Oropharynx	73 (91.2)	22 (30.1)	0.12 (NS)
Non‐oropharyngeal	7 (8.8)	4 (57.1)	
Tumour stage^c^			
I	20 (26.7)	4 (20)	**0.002**
II	32 (42.7)	7 (21.9)	
III	22 (29.3)	12 (54.5)	
IV	1 (1.3)	1 (100)	
Lymph node invasion^c^			
Yes	64 (85.3)	21 (32.8)	0.68 (NS)
No	11 (14.7)	3 (27.3)	
HPV genotype			
Genotype 16	72 (90)	22 (30.5)	0.16 (NS)
Other genotypes	8 (10)	4 (50)	
Tumour differentiation^d^			
Well/moderate	32 (56.1)	14 (43.8)	0.36 (NS)
Poor	25 (43.9)	8 (32)	
Mitotic index^e^			
Low/moderate	8 (20)	3 (37.5)	0.93 (NS)
High	32 (80)	11 (34.4)	
Perineural invasion^f^			
Yes	12 (28.6)	4 (33.3)	0.97 (NS)
No	30 (71.4)	8 (26.7)	
Lymphovascular invasion^g^			
Yes	17 (37)	6 (35.3)	0.80 (NS)
No	29 (63)	10 (34.5)	
Initial therapy^a^			
Surgery with or without induction CT	8 (10.1)	1 (12.5)	0.55 (NS)
Exclusive (chemo)RT	15 (19)	5 (33.3)	
Surgery followed by (chemo)radiation	54 (68.4)	19 (35.2)	
None	2 (2.5)	1 (50)	

^a^ Data available for 79 patients.

^b^ Data available for 76 patients.

^c^ Data available for 75 patients.

^d^ Data available for 57 patients

^e^ Data available for 40.

^f^ Data available for 42.

^g^ Data available for 46.

^h^ Univariate analysis (Kaplan–Meier method).

### 3.2 HPV genotypes

HPV genotype, initially determined by PCR, was confirmed by the double Capture‐HPV method followed by NGS. HPV16 was the most common genotype (*n* = 72/80, 90%) (Table 1). The other HPV genotypes were HPV 33 (*n* = 4/80, 5%), HPV 35 (*n* = 2/80, 2.5%), HPV 26 (*n* = 1/80, 1.2%) and HPV 56 (*n* = 1/80, 1.2%). We did not find patients co‐infected with several HPV genotypes.

### 3.3 Integrations signatures

When determining viral integration status by comparing the mean depth of coverage at the E2 and E6 genes loci, we identified 3 groups of patients with episomal, integrated and mixed (Tables S1 and S2; Fig. S1). Thirty‐one patients harbored episomal HPV (*n* = 31/80, 38.8%), whereas 49 (*n* = 49/80, 61.2%) displayed HPV integration among whom only 6 patients had a pure integration (Tables S1 and S2). The ratio of E2/E6 expression was differential among episomal and integrated/mixed confirming that E2 expression level is lower in HPV integrated samples (Table S2).

**Fig. 1:**
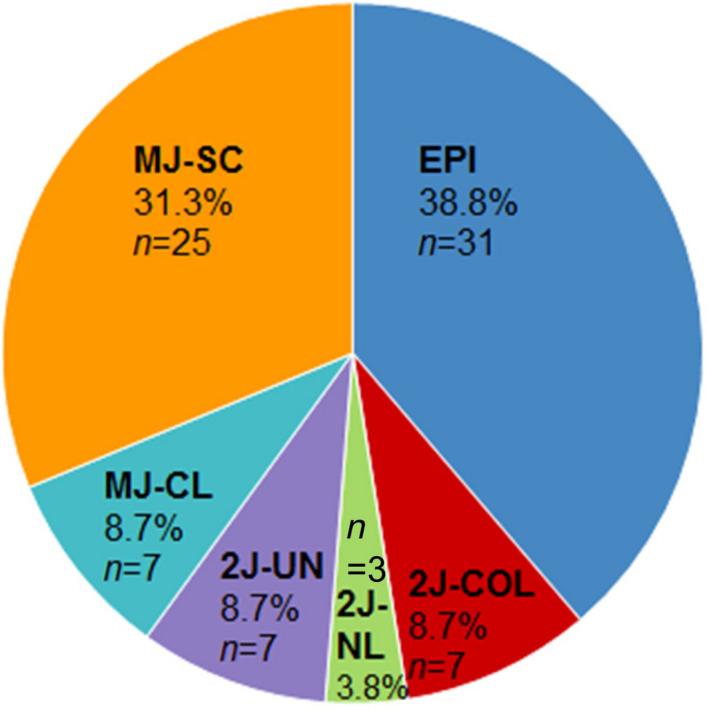
Distribution of HPV genomic signatures in 80 patients with HPV positive head and neck squamous cell carcinoma. 2J‐COL: two hybrid colinear junctions; 2J‐NL: two hybrid nonlinear junctions; 2J‐UN: two hybrid junctions with a lost junction; EPI: episomal; MJ‐CL: multiple hybrid junctions clustered in one locus; MJ‐SC: multiple hybrid junctions scattered at distinct loci.

In addition to the 31/80 episomal HPV, the mechanistic HPV genomic signatures of the 49 integrated and mixed HPV tumours, were MJ‐SC (*n* = 25/80, 31.3%), MJ‐CL (*n* = 7/80, 8.7%), 2J‐COL (*n* = 7/80, 8.7%), 2J‐NL (*n* = 3/80, 3.8%) and seven 2J‐UN (*n* = 7/80, 8.7%) (Fig. 1). The distribution of HPV signatures was statistically close to the one observed in our cohort of 93 ASCC (*P* = 0.94) (Fig. S2A) [15] but markedly different from that observed in our cohort of 272 CC (*P* < 0.0001) (Fig. S2B) [16].

### 3.4 Viral genes deletion

All patients displaying HPV integration exhibited deletion in some viral genes, albeit systematically retaining *E6* and *E7* oncogenes. Deletions were observed in *E1, E2, E5, L1* or *L2* viral genes (data not shown).

### 3.5 Patients with synchronous tumour samples

Both tumour and lymph node samples at diagnosis were available for two patients. HPV16 was the viral genotype in both cases. The signatures were the same at both sites (one patient with EPI and one with MJ‐SC). The Table S3 shows the breakpoints of the MJ‐SC patient. The four same breakpoints were found in the primary tumour and cervical nodes. However, four additional breakpoints were present in the primary tumour. Of note, two additional breakpoints (supported by respectively eight and five reads) were detected on the primary tumour and not on the lymph nodes samples.

### 3.6 Association between HPV genomic signatures and clinico‐biological characteristics

Table 2 presents the distribution of HPV genomic signatures (grouped into EPI, 2J and MJ) according to the patients’ clinical and pathological characteristics. We observed no significant association between HPV genomic signatures and characteristics (Table S4). However, we noted a trend towards an association between the EPI signature and absence of lymph node invasion (*n* = 7/29, 23.3% in the EPI population vs. *n* = 4/46, 8.7% in the HPV integrated population, *P* = 0.07) and for the absence of lymphovascular invasion (*n* = 11/14, 80% in the EPI vs. *n* = 18/32, 56.2% in the HPV integrated population, *P* = 0.14). We also found a trend for an association between the HPV signature and HPV copy number (*P* = 0.06). The association is statistically significant between 2J signature and low HPV copy number (*n* = 12, 70.6%), as compared to the “not 2J” population (*n* = 24, 38%) (*P* = 0.020).

**Table 2:** Association between HPV genomic signatures and clinical and pathological characteristics of 80 patients with HPV‐positive head and neck squamous cell carcinoma.

Heading	Patients (%)	Number of patients (%)			*P‐*value^h^
		EPI	2J	MJ	
Total	80 (100)	31 (38.8)	17 (21.2)	32 (40)	
Age (years)					0.51 (NS)
<65	45 (56.2)	15 (50)	10 (58.8)	20 (62.5)	
≥65	35 (43.8)	16 (50)	7 (41.2)	12 (37.5)	
Gender					0.44 (NS)
Male	60 (75)	23 (71.9)	11 (64.7)	26 (81.2)	
Female	20 (25)	8 (28.1)	6 (35.3)	6 (18.8)	
Tobacco^a^					0.76 (NS)
Yes	48 (60.8)	17 (58.1)	10 (58.8)	21 (65.6)	
No	31 (39.2)	13 (41.9)	7 (41.2)	11 (34.4)	
Alcohol^b^					0.44 (NS)
Yes	19 (25)	5 (20.7)	4 (23.5)	10 (33.3)	
No	57 (75)	23 (79.3)	13 (76.5)	21 (67.7)	
Localization					0.62 (NS)
Oropharynx	73 (91.2)	29 (93.8)	16 (94.1)	28 (87.5)	
Non‐oropharyngeal	7 (8.8)	2 (6.2)	1 (5.9)	4 (12.5)	
Tumour stage^c^					0.40 (NS)
I	20 (26.7)	9 (29.0)	4 (26.7)	7 (23.3)	
II	32 (42.7)	11 (35.5)	5 (33.3)	16 (53.3)	
III	22 (28.3)	10 (35.5)	5 (33.3)	7 (23.3)	
IV	1 (1.3)	0 (0)	1 (6.7)	0 (0)	
Lymph node invasion^c^					0.18 (NS)
Yes	64 (85.3)	22 (76.7)	14 (93.3)	28 (90.3)	
No	11 (14.7)	7 (23.3)	1 (6.7)	3 (9.7)	
HPV genotype					0.96 (NS)
Genotype 16	72 (90)	28 (90.6)	15 (88.2)	29 (90.6)	
Other genotypes	8 (10)	3 (9.4)	2 (11.8)	3 (9.4)	
Tumour differentiation^d^					0.33 (NS)
Well/moderate	32 (56.1)	14 (51.9)	5 (41.7)	13 (68.4)	
Poor	25 (43.9)	12 (48.1)	7 (58.3)	6 (31.6)	
Mitotic index^e^					0.41 (NS)
Low/moderate	8 (20)	5 (22.7)	0 (0)	3 (23.1)	
High	32 (80)	16(77.3)	6 (100)	10 (76.9)	
Perineural invasion^f^					0.32 (NS)
Yes	12 (28.6)	6 (37.5)	1 (11.1)	5 (27.8)	
No	30 (71.4)	9 (62.5)	8 (88.9)	13 (72.2)	
Lymphovascular invasion^g^					0.34 (NS)
Yes	17 (37)	3 (20)	4 (40)	10 (45.5)	
No	29 (63)	11 (80)	6 (60)	12 (54.5)	
Initial therapy^a^					0.32 (NS)
Surgery with or without induction CT	8 (10.1)	3 (9.7)	1 (5.9)	4 (12.5)	
Exclusive (chemo) RT	15 (19)	4 (12.9)	2 (11.8)	9 (28.1)	
Surgery followed by (chemo) radiation	54 (68.4)	21 (70.9)	14 (82.3)	19 (59.4)	
None	2 (2.5)	2 (6.5)	0	0	
HPV copy number					0.06 (NS)
Low (<9)	36 (45)	12 (40.5)	12 (70.6)	12 (37.5)	
High (≥9)	44 (55)	19 (59.5)	5 (29.4)	20 (62.5)	

^a^ Data available for 79 patients.

^b^ Data available for 76 patients.

^c^ Data available for 75 patients.

^d^ Data available for 57 patients.

^e^ Data available for 40.

^f^ Data available for 42.

^g^ Data available for 46.

^h^ Chi‐square test *P* (with Yates correction if appropriate) values for comparison of the EPI group vs. 2J group vs. the MJ group for each parameter; tumour stage UICC 8e classification CT: chemotherapy; RT: radiotherapy.

### 3.7 Integration sites

To validate suspected HPV integration into the human genome, we designed primers with one of them being derived from the human genome at the potential site of integration and the other against HPV sequences suspected of being near the site of integration within the HPV genome. The primers were designed 200–400 bp away from the detected integration sites based on the NGS reads.

**Fig. 2:**
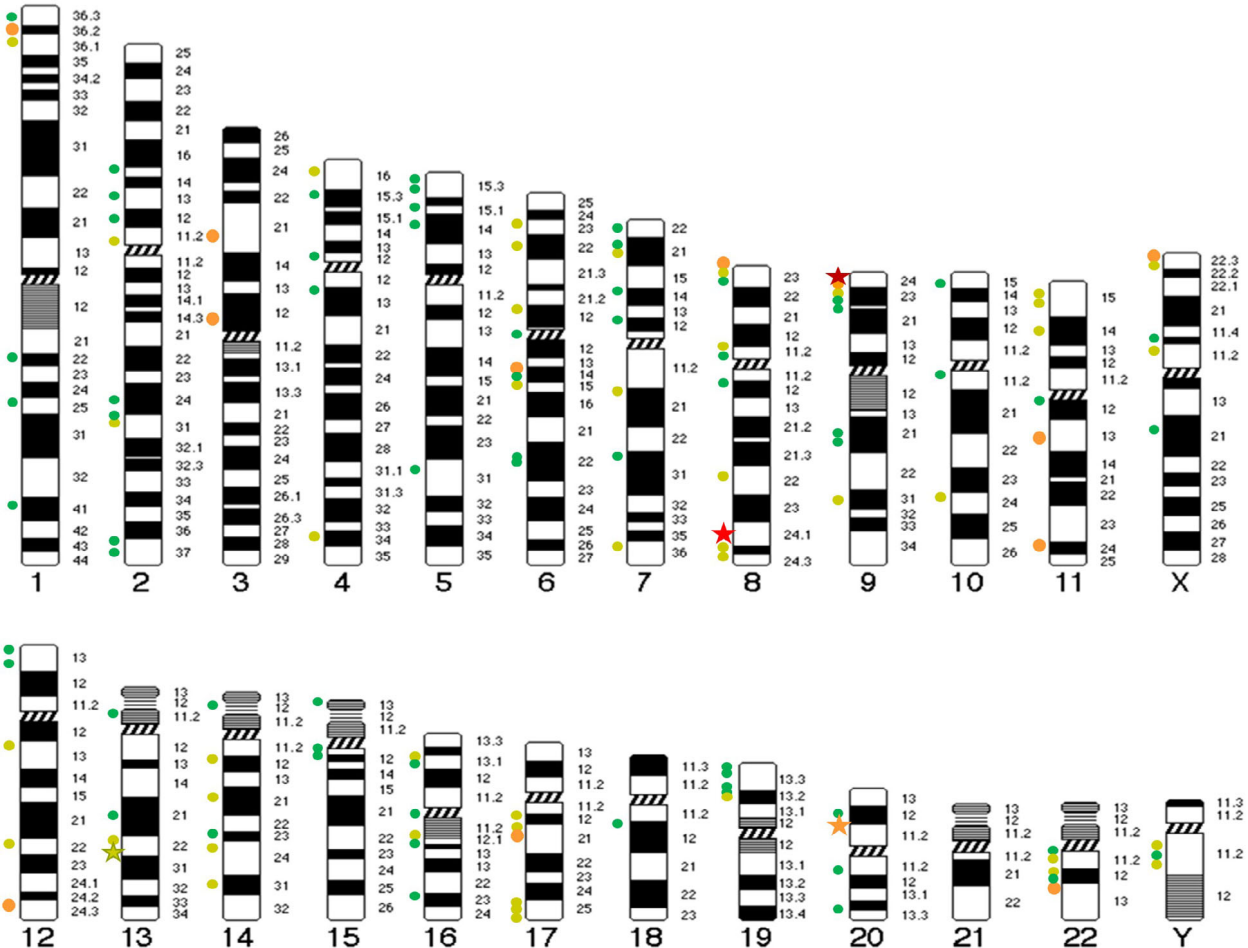
Chromosomal distribution of the 267 HPV‐human chromosome junction sequences found in 49 patients with HPV positive head and neck carcinoma. The orange dots represents 4 to 8 different breakpoints in the region, the light green dots 2 or 3 breakpoints, the dark green dots depict unique breakpoint in the region. The dark red star represents the 27 breakpoints in *PDL1* region, the light red star the 20 breakpoints in *MYC* region, the orange star represents the 5 breakpoints in *MACROD2* region and the light green star the 3 breakpoints in *KLF5* region.

Integration in the human genome was observed scattered on all chromosomes except on the small chromosome 21 as represented in Figure 2. We identified 267 HPV‐human chromosome junction sequences (Table S5) among the 49 tumours with HPV integration. We assessed the proportion of cancer‐related genes in the 267 HPV genome insertion sites (located ≤ 10^6^ bp from the integration site). The protein coding genes represent 141/267 genes and are enriched in genes related to cancer (based on 685 OncoKB genes) as compared to the total number of genes in a human genome (based on 19 982 GENCODE protein coding genes).

In fact, OncoKB cancer related genes represent 11.3% (16/141) of the protein coding genes found near insertion sites, as compared to 3.4% of the entire human genome (685/19 982) (Chi‐square test, *P* = 1.06 × 10^‐6^).

The number of HPV integration sites per tumour corresponds to 16.8 (267/49) in our series of HNSCC, while it is 5.5 for anal [15] and cervical cancers [16]. All integration sites were unique at the nucleotide level. Patients had at least one intragenic integration in 77.6% (*n* = 38/49, 77.6%), whereas 22.4% of patients only harbored intergenic integrations (*n* = 11/49, 22.4%). HPV‐Human DNA recombinations within a single sample (≥3 junctions) were detected for 15/49 HPV integrated patients in our cohort. PCR and sequence analyses confirmed the 7 junctions observed in sample R14 (Data not shown).

**Fig. 3:**
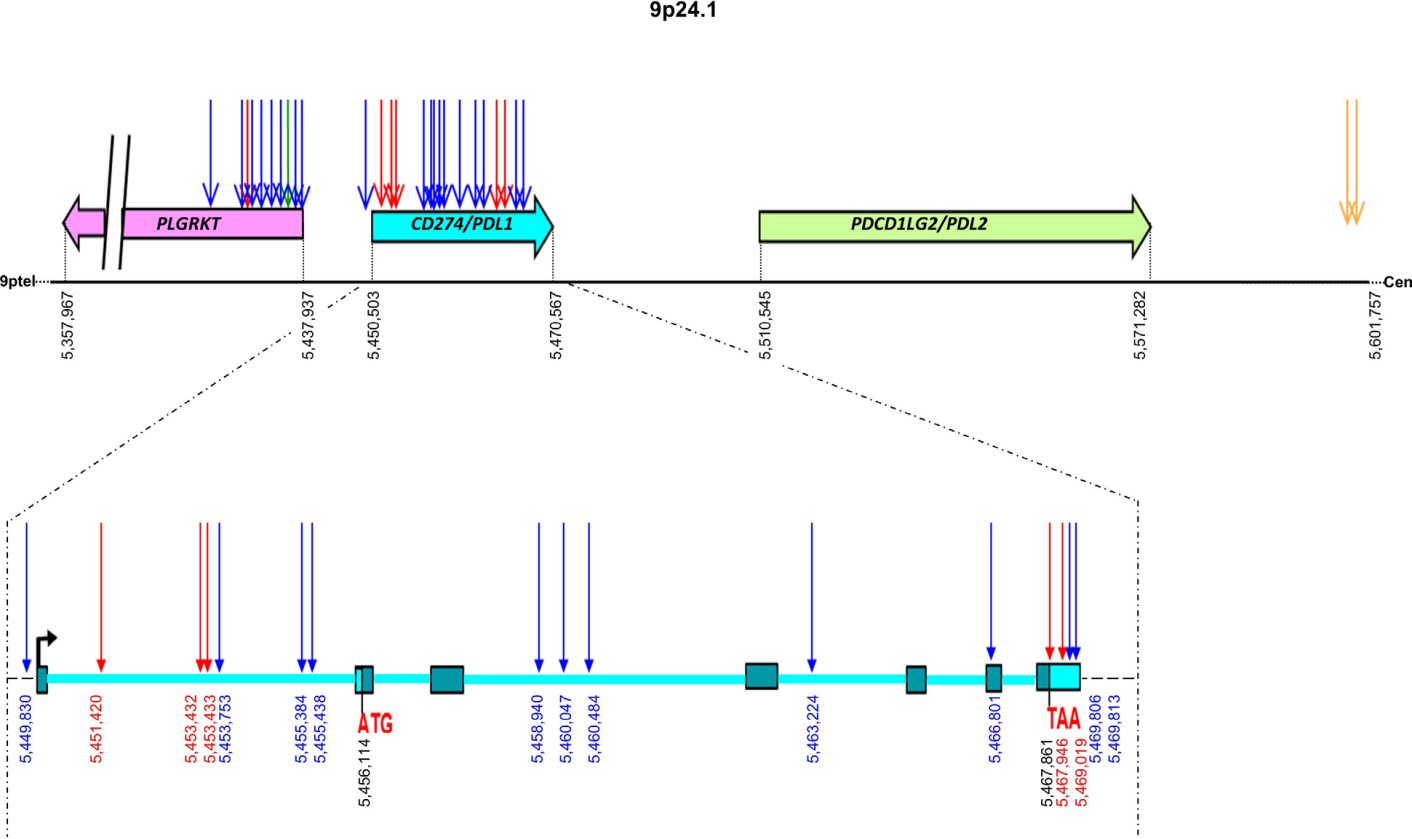
HPV integration breakpoints in the chromosomal region 9p24.1 in four patients with head and neck carcinoma. We represented *PDL1* 7 exons, the 3’ UTR and the 5’UTR with the starting codon (ATG) and the stop codon (TAA). Each color corresponds to a patient: R295, R299, R613 and R650. Each Arrow is a breakpoint position. 9ptel indicates the telomere and Cen the centromere. The numbers indicate the genomic positions.

Remarkably, we found four recurrent integration regions. The first chromosomal region with frequent integration (*n* = 4/49, 8.2%) was the 9p24.1 region. It contains two important immune checkpoint genes: *PDL1*, PDL2 and *PLGRKT* (Fig. 3). Most breakpoints happened into introns or in the 3’ or 5’UTR. One breakpoint was in the 6^th^ exon of *PDL1*. This exon is located at the 3’ end and after the sequence coding for the functional domain of PDL1 protein. Three patients (*n* = 3/49, 6.1%) had integration near *MYC* and *PVT1* in the 8q24.21 region (Fig. S3). *MACROD2* integration was found in two patients (*n* = 2/49, 4%). Finally, two patients (*n* = 2/49, 4%) had integration in the *KLF5/KLF12* containing region.

### 3.8 Signatures’ association with survival, copy number or integration region

No statistical difference was observed in terms of DFS (Fig. S4A–C) or OS (Fig. S4D–F) in patients with episomal, integrated and mixed HPV statuses. These results were confirmed in the oropharynx patients’ subpopulation (Fig. S4G–J). Additionally, no association was found between HPV genomic signatures and OS (*P* = 0.65) or DFS (*P* = 0.87) (Fig. S5A and C). There was a trend towards an association between HPV copy number and survival: patients with a high HPV copy number had longer OS (*P* = 0.08) and DFS (*P* = 0.07) (Fig. S5B and D). No association was found between survival and the recurrent integration hotspots (data not shown).

### 3.9 mRNA expression of HPV targeted genes

**Fig. 4:**
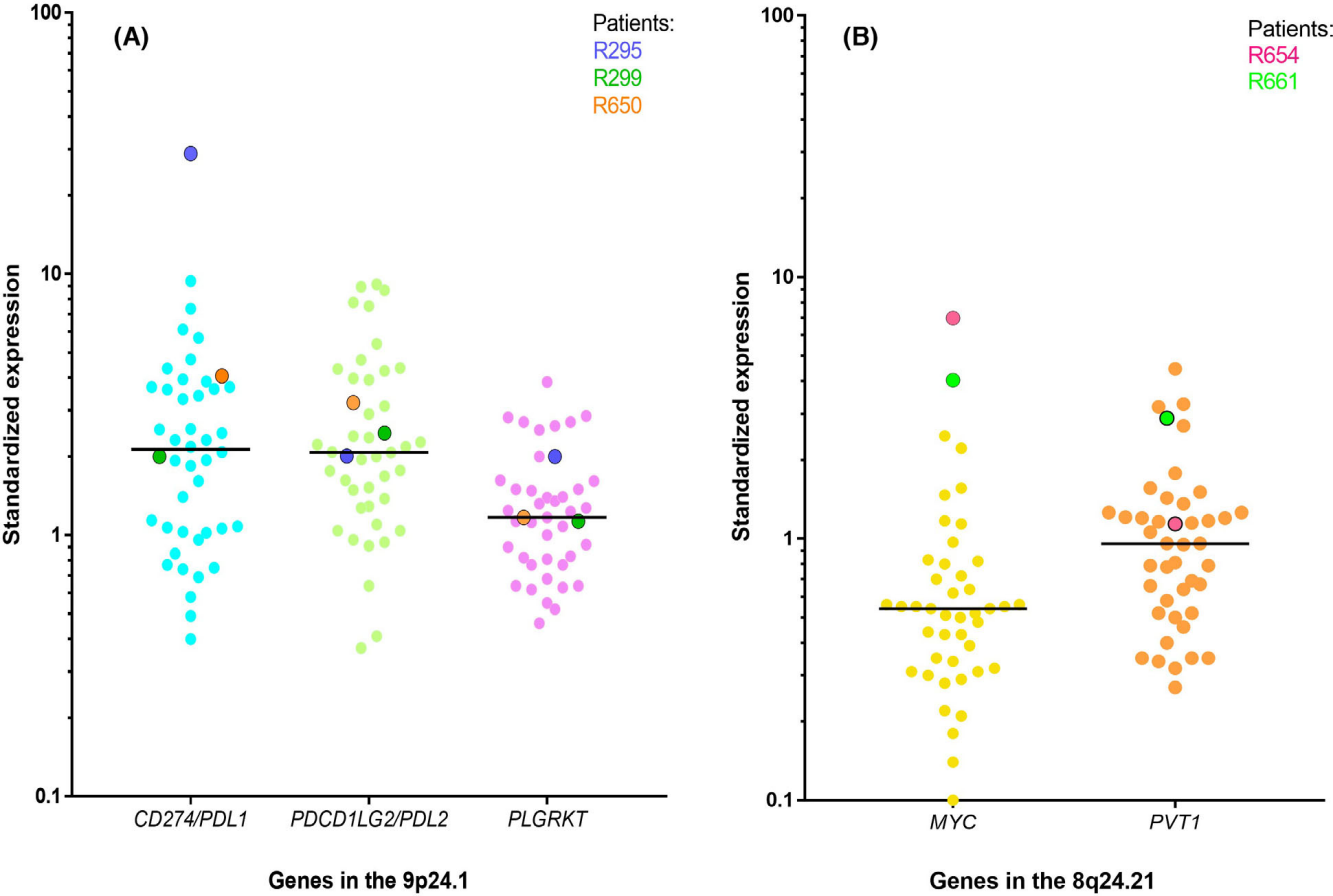
mRNA expression level of *PDL1, PDL2* and *PLGRKT* (A) and *MYC* and *PVT1* (B) and in 44 patients with HPV positive head and neck squamous cell carcinoma. Each dot represents a patient. The horizontal bars display the median for each data set. (A) Patients R295, R299 and R650 have integration in the 9p24.1 chromosomal region. (B) Patients R654 and R661 have integration in the 8q24.21 chromosomal region.

Total RNA was available for three (R295, R299 and R650) of the four patients with HPV integration in the *PDL1/PDL2/PLGRKT* region, and for two (R654 and R661) of the three patients with HPV integration in the *MYC/PVT1* region. Figure 4 represents the targeted gene expressions of the 44 HNSCC patients with available RNA. One patient (R295) among the three patient with integration in the *PDL1, PDL2* and *PLGRKT* containing region showed overexpression of *PDL1* (but not of *PDL2* and *PLGRKT)* (Fig. 4A). R295 is the only tumour with HPV integration in the promoter region of *PDL1* (Fig. 3 and Fig. 4A). *MYC* (but also *PVT1* at a lower level) is overexpressed in the two patients with HPV integration in the *MYC/PVT1* region, as compared to the expression in the 42 other HNSCC tumours (Fig. 4B).

### 3.10 Relationship between HPV genomic signatures and mRNA expression of immune‐related genes

We tested the possible association between HPV genomic signatures and the expression of various immune‐related genes in our cohort of 44 HNSCC patients with available RNA (Table S6). We observed a significant association between HPV genomic signature and the fibroblast marker *PDGFRß* (*P* = 0.04). There were trends towards an association between 2J signature and higher expression (near two fold as compared to the two other groups) of several genes *CD20* (marker of B lymphocytes), *CD163* (marker of M2 macrophages), *ITGAM* (marker of myeloid‐derived suppressor cells), as well as *IL8* (neutrophils chemoattractant cytokine), *OX40L* (a major immune checkpoint), *VIM* (marker of EMT) and *APOBEC3H* but not *APOBEC3A* and *APOBEC3B*. Of note, there was no association between HPV genomic signature and well‐known markers of cell proliferation such as *MKI6* or *CCND1*.

## 4 Discussion

In the present study, we used the robust and sensitive double capture method, followed by NGS on 80 patients with HPV positive HNSCC. HPV16 was the main detected genotype. No HPV co‐infection was noted. We did not find any HPV18 infection. We confirmed the five previously described mechanistic HPV signatures with distribution close to the one observed in ASCC but different from the one described in CC. No correlation was established between HPV genomic signature s and known clinico‐biologic prognostic factors. A low HPV copy number tended to be associated with worse prognostic and with the 2J signature. We identified 267 HPV‐human chromosome junction sequences scattered on all chromosomes with four integration hotspot regions (*PDL1* region, *MYC* and *PVT1* region, *MACROD2* region and in *KLF*5/*KLF12* region). *PDL1* and *MYC* were overexpressed upon integration.

The HPV16 predominance and the absence of HPV18 could be explained by the high proportion of oropharyngeal tumour in our samples. HPV18 is known to be rare in HPV‐positive oropharyngeal SCCs compared with other sites (34% of oral and 17% of laryngeal SCCs) [6]. In most cases, the HPV genomic signature was EPI then it was MJ‐SC, followed by MJ‐CL, 2J‐COL and other 2J‐UN. The distribution of HPV signatures was statistically consistent with the one observed in ASCC but very different from the one described in CC [15, 16]. HPV remained episomal in 38.8% of patients with HNSCC, 45% in ASCC, whereas only 12% in CC. One explanation might be HPV genotypes repartition between cancer types. HPV16 is the major genotype in ASCC and HNSCC, and this particular genotype displays more EPI signature. Holmes et al. [14] reported HPV18 to display more integration whereas HPV16 genotypes exhibited more EPI signature. The second explanation could be that HPV integration plays a bigger role in the carcinogenesis of CC than in HNSCC and ASCC [16]. The integration rate increases with the degree of transformation from normal epithelium to cervical intraepithelial neoplasia to invasive cancers with an integration rate up to 90% [17] ^(p)^. Further studies using long read sequencing such as Pacific Biosciences (PacBio) and Oxford Nanopore Technologies (ONT) are needed to (a) assess the mechanistic of HPV insertion and junction‐related signatures and (b) to differentiate between hybrid human–virus ecDNA and chromosomal HPV DNA [24, 29] in our 61.2% integrated/mixed HPV patients.

HPV genomic signatures seem to have no impact on outcome or response to treatment. Previous studies found that HNSCC harbouring HPV integration had worse survival than those with HPV remaining in an episomic state [18]. We did not confirm this result, possibly due to our small sample size. In ASCC and CC, HPV copy number (also called “viral load” in other articles) was an independent prognostic factor [15, 16]. In our HNSCC cohort, patients with a high HPV copy number had a better prognosis. The small size of our cohort did not allow reaching statistical significance (*P* = 0.07). A recent study on HPV‐positive oropharyngeal tumours used digital droplet PCR to assess the copy number and confirmed that high viral copy number is associated with better clinical outcomes [39]. A possible explanation is that carcinogenesis is primarily virus driven in samples with high copy number, whereas cancers with low copy number are driven by other oncogenic events. We found a trend to significance for an association between HPV genomic signatures and HPV copy number (*P* = 0.06). The association is statistically significant between 2J signature and low HPV copy number when compared to the “not 2J” population (*P* = 0.020).

Two patients with matched samples (primary tumour and synchronous lymph nodes) displayed the same signatures at both sites. This finding supports the data on HPV clonal evolution during HPV induced carcinogenesis [40]. In the MJ‐SC patient, we found the four same breakpoints in the primary tumour and cervical nodes. However, four additional breakpoints were only present in the primary tumour sample. We explain these results by the difference of sequencing coverage between the two samples (5‐fold variation in the number of reads).

Conversely to cervical cancer, few studies documented in the literature report HPV integration sites in HNSCC. We observed 267 HPV‐human chromosome junction sequences scattered on human chromosomes. We identified four chromosomal regions with recurrent HPV integration. The first hotspot integration region, observed in 8.2% of our patients, is the 9p24.1 chromosomal region. It includes three genes: *PDL1*, *PDL2* and *PLGRKT*. *PDL1* and *PDL2* encode for the two ligands for the PD‐1 immune inhibiting checkpoint, which plays a role in the negative regulation of the adaptive immune response. PD‐L1 or PD‐L2 are upregulated in many human tumours, including HNSCCs [41]. High level of PD‐L1 favors immune escape. We observed *PDL1 (CD274)* amplification (Fig. S6A) and overexpression upon integration. The patient with the highest PDL1 expression has a breakpoint in the promotor region leading to gene amplification and overexpression. HPV integration in this hotspot region is described in other studies, also resulting in an overexpression of *PDL1* [18]. Immune checkpoint inhibitors targeting the PD1‐PDL1 axis are approved for the treatment of recurrent and/or metastatic HNSCC [3, 4, 5]. Exploratory analyses of Checkmate‐141 (nivolumab) and Keynote‐012 (pembolizumab) clinical trials, suggest a greater benefit of immune checkpoints inhibitors in patients with PDL1 status ≥1% as well as in p16 positive patients [3, 42]. Further studies are needed to verify if HPV disruption of *PDL1* or *PDL2* is a predictive marker of response to PD1‐PDL1 targeting therapy in HNSCC.

The second HPV integration hotspot is the region containing *MYC* and *PVT1*, 6.1% of patients had integration in the region. *MYC* is a well‐known oncogene that code for a transcription factor regulating the expression of 15% of human genes [16]. *PVT1* is a long non‐coding RNA (lncRNA) showing aberrant expression in multiple human cancer types. *PVT1* is implicated in the malignant progression of HNSCC and represents a potential biomarker and therapeutic target in HNSCC [43] ^(p1)^. It is known to interact with its neighbor *MYC*, resulting in *MYC* upregulation [44]. Several studies aiming to discover viral integration sites in the genome of host cells have demonstrated frequent integrations in the *MYC* gene in CC [14, 17, 25]. In our study, the patients with integration in the *MYC* region display the highest *MYC* expression, which may be explained by DNA focal gain (Fig. S6B). New therapeutic strategies are emerging in malignancies displaying *MYC* activation, for instance, with BET bromodomain inhibition [45].

The third HPV integration hotspot is *MACROD2*, with two patients displaying intragenic HPV integration. The protein encoded by this gene is a deacetylase involved in removing ADP‐ribose from mono‐ADP‐ribosylated proteins; it has a key role in DNA repair. Our team previously identified *MACROD2* to be a hotspot of HPV integration in CC [16]. *MACROD2* loss has already been described in colorectal and hepatocellular carcinogenesis [46, 47]. *MACROD2* is a caretaker tumour suppressor gene. Deletions alter DNA repair and sensitivity to DNA damage thought impaired PARP1 activity resulting in chromosome instability [47, 48].

The last recurrent HPV integration hotspot was the chromosomal region 13q22.1 where are located *KLF5* and *KLF12* genes, identified in two patients. These genes along with *MYC* are already described as HPV integration hotspot in cervical cancer [14, 25]. We observed an additional tumour with integration in *KLF14* located in 7q32.3, one in *KLF4* located in 9q31.2, and one in *KLF6* located in 10p15.1 (Table S5). The Krüppel Like Factors (KLF) refers to a family of seventeen members of transcription factors with key functions in many cellular processes: proliferation, differentiation, migration, inflammation and pluripotency [49]. Deregulation of the KLF has been shown in HNSCC [50].

Several studies suggested HPV modulation of local immunity in various cancers [20]; therefore, we performed gene expression analysis on key immune related genes. We observed a significant association between HPV genomic signature and the fibroblast marker *PDGFRß*. No other genes were associated with HPV genomic signatures. This might be explained by our small sample size and would require a more dedicated study, that in addition was based on selected genes, which are not fully specific of each immune cell subtypes.

This work provides new insights into the role of HPV integration in HNSCC and opens new avenues regarding the biological interplay between the virus and HNSCC immune microenvironment. Our study has several limitations: First of all, the small sample size might have hindered reaching statistical significance when correlating to clinical outcome or when assessing the relationship between HPV genomic signatures and immune‐related gene expressions analyses. A second limitation is the long inclusion period (1997–2017) when cancer treatments have consistently evolved over the past twenty years. Dedicated functional analyses are needed to better evaluate HPV integration consequences on immune microenvironment. Overall, prospective clinical studies on HPV‐positive HNSCC patients need to be conducted to demonstrate the antitumour efficacy of targeting agents in light of the molecular alterations identified as companion biomarkers (*PDL1* and *MYC*) in the present study.

## 5 Conclusion

For many years, HPV oncogenic potential was only attributed to the viral oncoproteins E6 and E7, but recent studies highlights that HPV integration is an oncogenic event *per se*. Here, we described recurrent HPV integration in four chromosomal regions *(*the *PDL1* region, *MYC* and *PVT1* region, *MACROD2* region and *KLF5* and *KLF12* region). *PDL1* and *MYC* genes were overexpressed upon HPV integration. Our hotspot genes have a direct role in carcinogenesis with different mechanisms: immunomodulation, loss of tumor suppressor, activation of oncogenes and upregulation of transcription factors. HPV induced HNSCC carcinogenesis is more complex than first described. It relies on three actors: the virus, the host and the immune system.

## Conflict of interest

All other authors report no conflict of interest.

## Author contributions

Conception and design: IB, CLT and, MK. Development of methodology: IB, CLT, NS, SL and AN. Acquisition of data: CC, JPD, MK, JM, AM, ES, PS, AS, SV, JK, SI, SL, JMP and ADV. Analysis and interpretation of data (e.g., statistical analysis, biostatistics and computational analysis): MD, JM, NS, SV, EG, AH and JMP. Writing, review and/or revision of the manuscript: JM, IB, CLT, MK, CD, LLC, CL and RR. Study supervision: IB and MK.

### Peer review

The peer review history for this article is available at https://publons.com/publon/10.1002/1878‐0261.13219.

## Supporting information


**Fig. S1A**. Samples analysis description and proportion of episomal, mixed and integrated HPV samples.

**Fig. S1B**. Variations of the depth of coverage at the E2 and E6 genes loci according to viral integration status.

**Fig. S2**. Distribution of HPV genomic signatures in patients with anal squamous cell carcinoma (A) or cervical cancer (B) and comparison to the signature observed in HNSCC. Chi‐square test, *P* value for global comparison of EPI group vs. MJ‐SC vs. MJ‐CL vs. 2J‐COL group vs. 2J‐NL vs. 2J‐UN between HNSCC vs. ASCC *P* = 0.94 and HNSCC vs. CC *P* < 0.0001. 2J‐COL: two hybrid colinear junctions; 2J‐NL: two hybrid nonlinear junctions; 2J‐UN: two hybrid junctions with a lost junction; EPI: episomal; MJ‐CL: multiple hybrid junctions clustered in one locus; MJ‐SC: multiple hybrid junctions scattered at distinct loci.

**Fig. S3**. HPV integration breakpoints in the chromosomal region 8q24.21 in three patients with HNSCC. We represented *PVT1* 9 exons. Each color corresponds to a patient: R654, 619 and R661. Each Arrow is a breakpoint position. 9ptel indicates the telomere and Cen the centromere. The numbers indicate the genomic positions.

**Fig. S4**. Survival curves representing the association between disease free survival (A–C) or overall survival (D–F) and HPV status in head and neck squamous cell carcinoma. Survival curves representing the association between disease free survival (G and H) or overall survival (I and J) and HPV status in oropharynx patients. No statistical difference was observed in terms of DFS (A–C) or OS (D–F) in patients with episomal, integrated and mixed HPV statuses. These results were confirmed in the oropharynx patients (G–J). Log‐rank test was used.

**Fig. S5**. Association with survivals in 80 patients with head and neck squamous cell carcinoma: (A) Association between disease free survival and HPV genomic signatures. (B) Association between disease free survival and HPV copy number. (C) Association between overall survival and HPV genomic signatures. (D) Association between overall survival and HPV copy number. EPI: episomal, 2J: two junction, MJ: multiple junctions, NS: non‐significant. Log‐rank test was used.

**Fig. S6**. Examples of focal gain or amplification of HPV integrated CD274 (A) and MYC (B) genes. Each arrow represents a focal gain or an amplification.

**Table S1**. Clonal breakpoints found in the primary tumour and in the lymph nodes of the same patient at diagnosis.

**Table S2**. HPV integration sites in human chromosomes of 49 HNSCC patients.

**Table S3**. Clonal breakpoints found in the primary tumour and in the lymph nodes of a same patient at diagnosis.

**Table S4**. Association between HPV genomic signatures and clinical and pathological characteristics of 80 patients with HPV‐positive head and neck squamous cell carcinoma.

**Table S5**. HPV integration sites in human chromosomes of 49 HNSCC patients.

**Table S6**. Associations between HPV genomic signatures and mRNA expression levels of immune‐related genes, EMT genes, cell proliferation genes and APOBEC genes.

## Data Availability

Nextflow‐based Virus Insertion Finder bioinformatics pipeline (v1.0.1) is available on free public access: https://github.com/bioinfo‐pf‐curie/nf‐VIF.
