# Supplementary figures and images for: Human papilloma virus integration sites and genomic signatures in head and neck squamous cell carcinoma
Source: Mol Oncol. 2022 May 10;16(16):3001–16. doi: 10.1002/1878-0261.13219 (PMC9394244; doi:10.1002/1878-0261.13219)

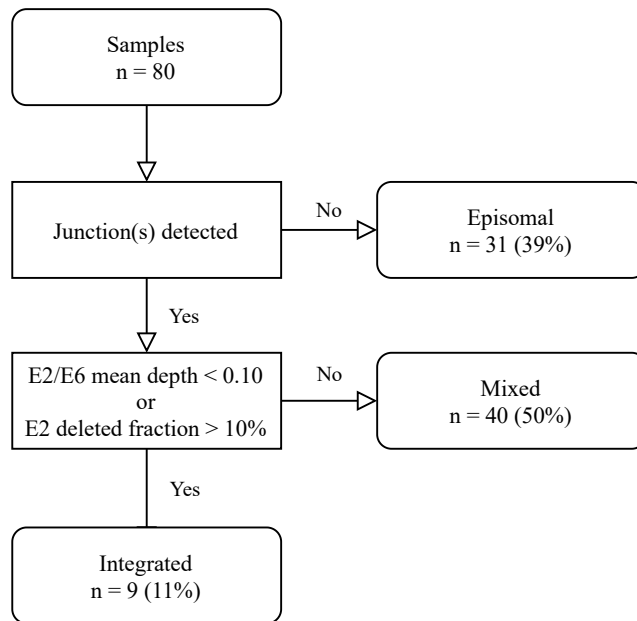

Supplement: Supplementary file 1 — Fig. S1A. Samples analysis description and proportion of episomal, mixed and integrated HPV samples. [file MOL2-16-3001-s001.pdf]

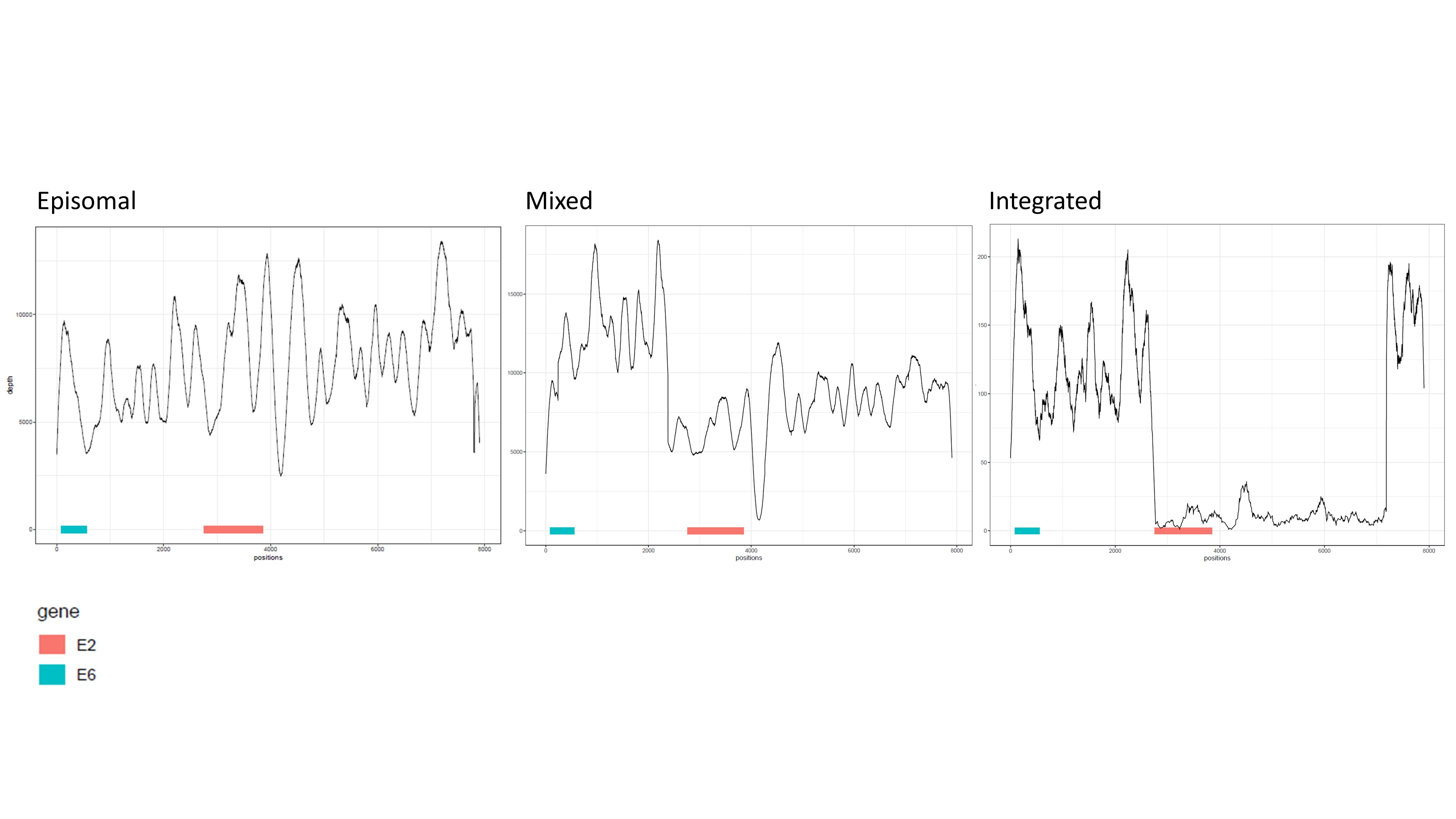

Supplement: Supplementary file 2 — Fig. S1B. Variations of the depth of coverage at the E2 and E6 genes loci according to viral integration status. [file MOL2-16-3001-s009.jpg]

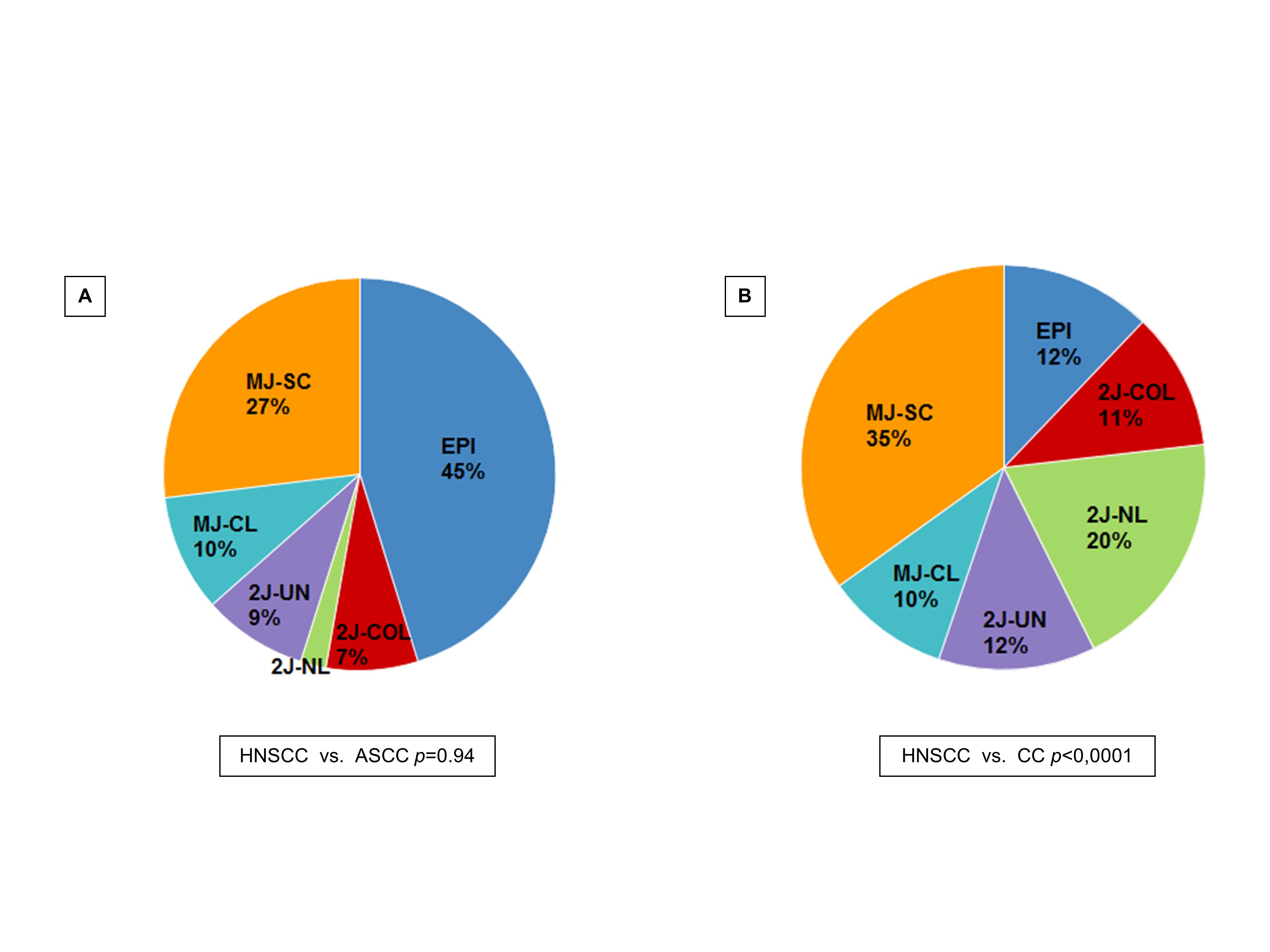

Supplement: Supplementary file 3 — Fig. S2. Distribution of HPV genomic signatures in patients with anal squamous cell carcinoma (A) or cervical cancer (B) and comparison to the signature observed in HNSCC. Chi‐square test, P value for global comparison of EPI group vs. MJ‐SC vs. MJ‐CL vs. 2J‐COL group vs. 2J‐NL vs. 2J‐UN between HNSCC vs. ASCC P = 0.94 and HNSCC vs. CC P < 0.0001. 2J‐COL: two hybrid colinear junctions; 2J‐NL: two hybrid nonlinear junctions; 2J‐UN: two hybrid junctions with a lost junction; EPI: episomal; MJ‐CL: multiple hybrid junctions clustered in one locus; MJ‐SC: multiple hybrid junctions scattered at distinct loci. [file MOL2-16-3001-s007.jpg]

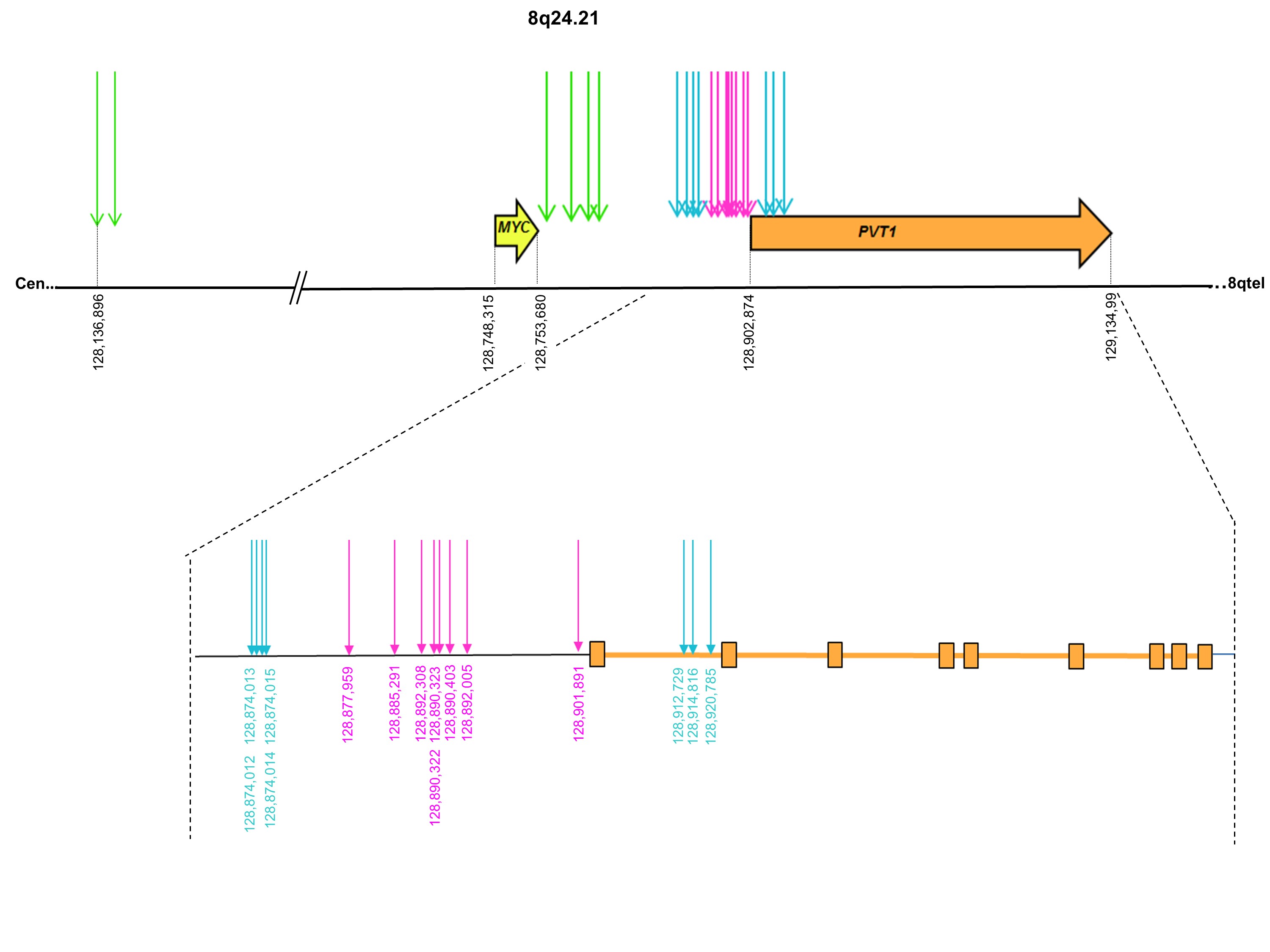

Supplement: Supplementary file 4 — Fig. S3. HPV integration breakpoints in the chromosomal region 8q24.21 in three patients with HNSCC. We represented PVT1 9 exons. Each color corresponds to a patient: R654, 619 and R661. Each Arrow is a breakpoint position. 9ptel indicates the telomere and Cen the centromere. The numbers indicate the genomic positions. [file MOL2-16-3001-s011.jpg]

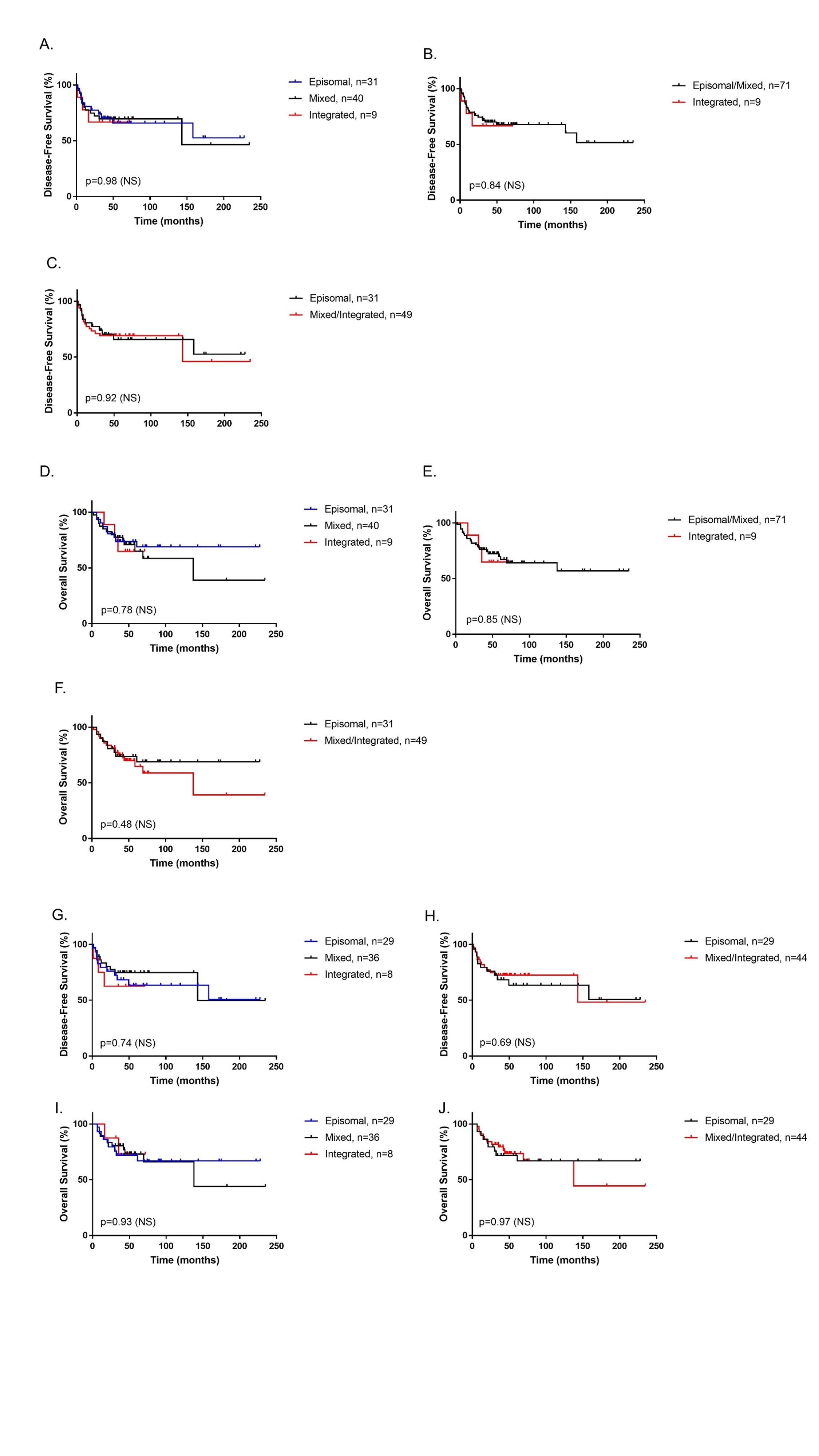

Supplement: Supplementary file 5 — Fig. S4. Survival curves representing the association between disease free survival (A–C) or overall survival (D–F) and HPV status in head and neck squamous cell carcinoma. Survival curves representing the association between disease free survival (G and H) or overall survival (I and J) and HPV status in oropharynx patients. No statistical difference was observed in terms of DFS (A–C) or OS (D–F) in patients with episomal, integrated and mixed HPV statuses. These results were confirmed in the oropharynx patients (G–J). Log‐rank test was used. [file MOL2-16-3001-s012.jpg]

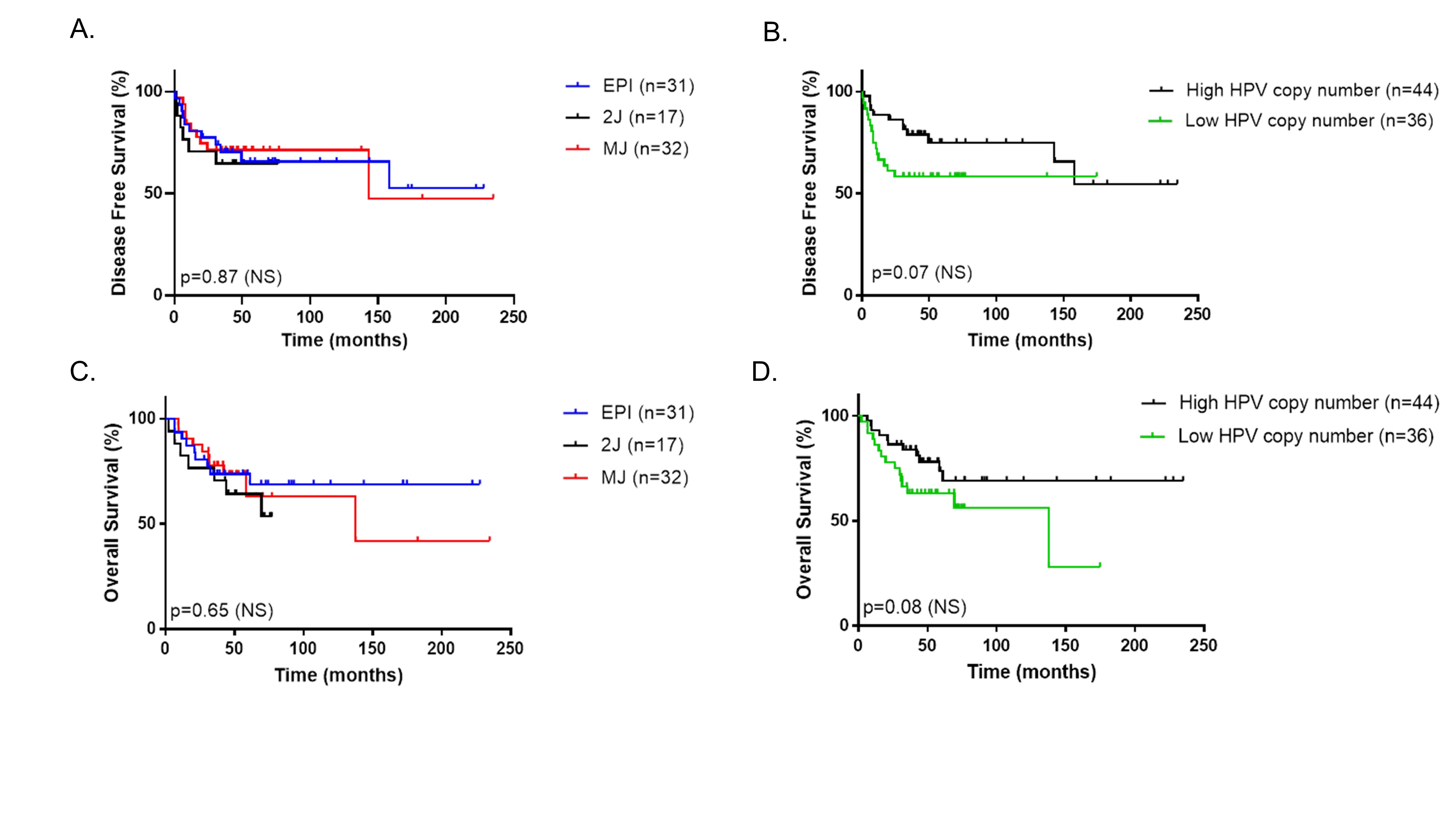

Supplement: Supplementary file 6 — Fig. S5. Association with survivals in 80 patients with head and neck squamous cell carcinoma: (A) Association between disease free survival and HPV genomic signatures. (B) Association between disease free survival and HPV copy number. (C) Association between overall survival and HPV genomic signatures. (D) Association between overall survival and HPV copy number. EPI: episomal, 2J: two junction, MJ: multiple junctions, NS: non‐significant. Log‐rank test was used. [file MOL2-16-3001-s002.jpg]

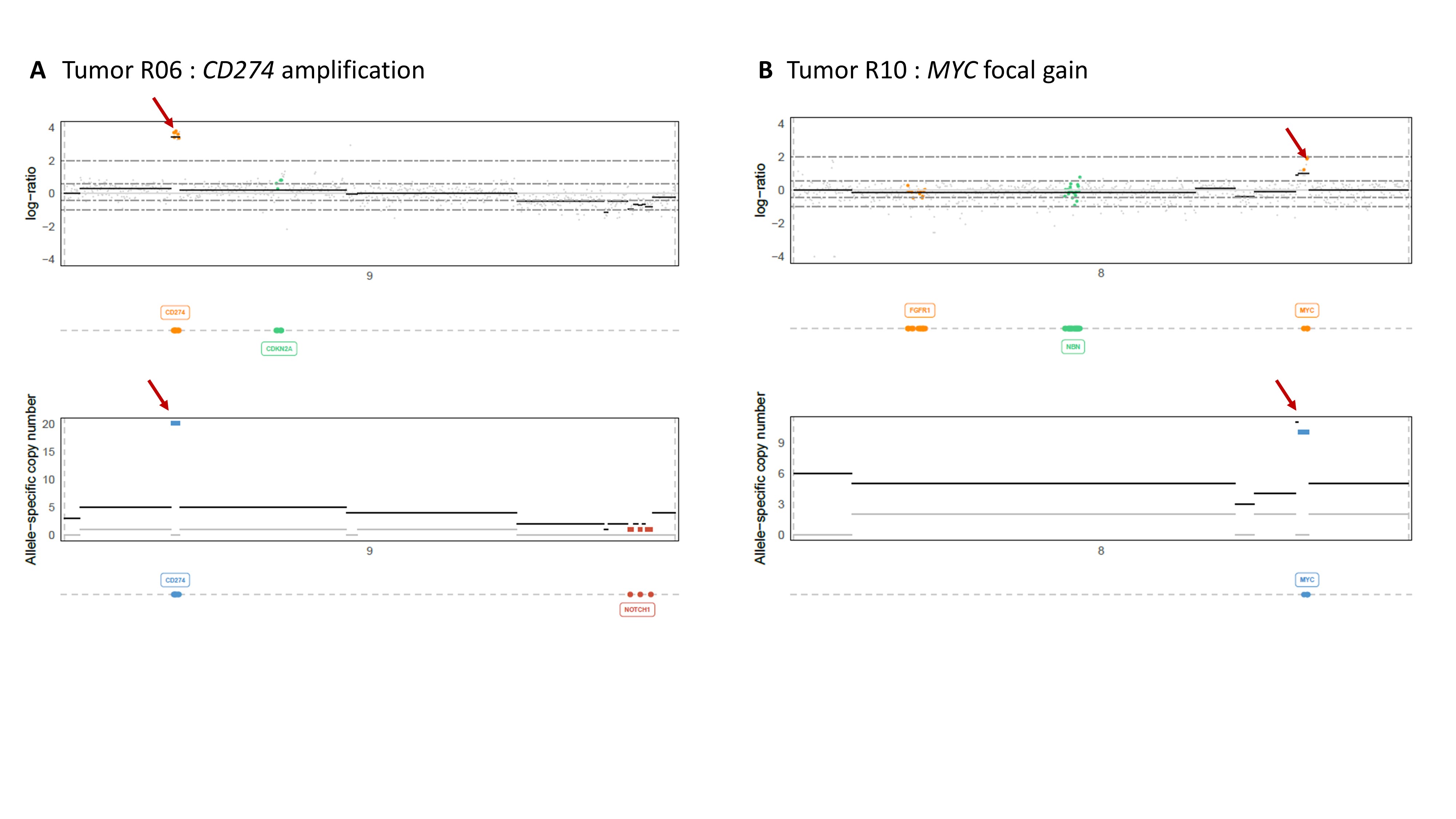

Supplement: Supplementary file 7 — Fig. S6. Examples of focal gain or amplification of HPV integrated CD274 (A) and MYC (B) genes. Each arrow represents a focal gain or an amplification. [file MOL2-16-3001-s013.jpg]
