# Supplementary material for: Human papilloma virus integration sites and genomic signatures in head and neck squamous cell carcinoma
Source: Mol Oncol. 2022 May 10;16(16):3001–16. doi: 10.1002/1878-0261.13219 (PMC9394244; doi:10.1002/1878-0261.13219)
Supplement: Supplementary file 10 — Table S3. Clonal breakpoints found in the primary tumour and in the lymph nodes of a same patient at diagnosis. [file MOL2-16-3001-s003.docx]

**Supplementary Table 3.** Clonal breakpoints found in the primary tumor and in the lymph nodes of a same patient at diagnosis.

| **Sample** | **HPV position** | **chromosome** | **Breakpoints*** | **read ends*** | **Nb reads /HPV** | **Total Ctrl genes** |
| --- | --- | --- | --- | --- | --- | --- |
| **Lymph nodes** | 3,237 | chr17 | 70,619,437 | 70,619,484 | 10,913 | 27,850 |
|  | 3,923 | chr17 | 70,619,523 | 70,619,459 |  |  |
|  | 2,666 | chr13 | 26,152,410 | 26,152,357 |  |  |
|  | 1,784 | chr12 | 53,994,655 | 53,994,568 |  |  |
| **Primary tumor** | 3,923 | chr17 | 70,619,523 | 70,619,450 | 39,821 | 173,999 |
|  | 3,237 | chr17 | 70,619,437 | 70,619,511 |  |  |
|  | 1,784 | chr12 | 53,994,655 | 53,994,571 |  |  |
|  | 2,666 | chr13 | 26,152,410 | 26,152,342 |  |  |
|  | 3,900 | chr2 | 162,179,283 | 162,179,255 |  |  |
|  | 2,111 | chr6 | 157,229,692 | 157,229,739 |  |  |
|  | 1,124 | chr2 | 92,316,071 | 92,315,988 |  |  |
|  | 7,046 | chr5 | 20,245,815 | 20,245,786 |  |  |

chr: chromosome

HPV position: position of the junction on the HPV genome

Breakpoints: position of the junction on the human genome

Nb reads/HPV: total number of sequenced HPV reads

Total Ctrl genes: total number of sequenced human control genes

*The human sequence version used is GRCh37
