# Supplementary material for: Human papilloma virus integration sites and genomic signatures in head and neck squamous cell carcinoma
Source: Mol Oncol. 2022 May 10;16(16):3001–16. doi: 10.1002/1878-0261.13219 (PMC9394244; doi:10.1002/1878-0261.13219)
Supplement: Supplementary file 11 — Table S4. Association between HPV genomic signatures and clinical and pathological characteristics of 80 patients with HPV‐positive head and neck squamous cell carcinoma. [file MOL2-16-3001-s006.docx]

**Supplementary Table 4. Association between HPV genomic signatures and clinical and pathological characteristics of 80 patients with HPV-positive head and neck squamous cell carcinoma**

| **Heading** | **Patients (%)** | **Number of Patients (%)** | | | | | | ***p-*value ^h^** |
| --- | --- | --- | --- | --- | --- | --- | --- | --- |
|  |  | **EPI** | **2J-COL** | **2J-NL** | **MJ-CL** | **MJ-SC** | **Other** |  |
| Total | 80 (100) | 31 (38.8) | 7 (8.8) | 3 (3.8) | 7 (8.8) | 25 (31.3) | 7 (8.8) |  |
| **Age (years)**  <65  ≥65 | 45 (56.2)  35 (43.8) | 15 (50)  16 (50) | 4 (57.1)  3 (42.9) | 2 (66.7)  1 (33.3) | 4 (57.1)  3 (42.9) | 16 (64)  9 (36) | 4 (57.1)  3 (42.9) | 0.91 (NS) |
| **Gender**  Male  Female | 60 (75)  20 (25) | 23 (71.9)  8 (28.1) | 5 (71.4)  2 (28.6) | 2 (66.7)  1 (33.3) | 6 (85.7)  1 (14.3) | 20 (80)  5 (20) | 4 (57.1)  3 (42.9) | 0.83 (NS) |
| **Tobacco^a^**  Yes  No | 48 (60.8)  31 (39.2) | 17 (58.1)  13 (41.9) | 4 (57.1)  3 (42.9) | 1 (33.3)  2 (66.7) | 4 (57.1)  3 (42.9) | 17 (68)  8 (32) | 5 (71.4)  2 (28.6) | 0.83 (NS) |
| **Alcohol^b^**  Yes No | 19 (25)  57 (75) | 5 (20.7)  23 (79.3) | 1 (14.3)  6 (85.7) | 1 (33.3)  2 (66.7) | 2 (28.6)  5 (71.4) | 8 (33.3)  16 (66.7) | 2 (28.6)  5 (71.4) | 0.81 (NS) |
| **Localization** Oropharynx  Non-oropharyngeal | 73 (91.2) 7 (8.8) | 29 (93.8) 2 (6.2) | 7 (100)  0 (0) | 3 (100)  0 (0) | 6 (85.7)  1 (14.3) | 22 (88)  3 (12) | 6 (85.7)  1 (14.3) | 0.84 (NS) |
| **Tumor stage^c^**  I  II  III  IV | 20 (26.7)  32 (42.7)  22 (28.3)  1 (1.3) | 9 (29.0)  11 (35.5)  10 (35.5)  0 (0) | 2 (28.6)  3 (42.9)  2 (28.6)  0 (0) | 1 (50)  0 (0)  1 (50)  0 (0) | 2 (33.3)  3 (50)  1 (16.7)  0 (0) | 5 (20.8)  13 (54.2)  6 (25)  0 (0) | 1 (16.7)  2 (33.3)  2 (33.3)  1 (16.7) | 0.40 (NS) |
| **Lymph node invasion^c^**  Yes  No | 64 (85.3)  11 (14.7) | 22 (76.7)  7 (23.3) | 7 (100)  0 (0) | 2 (100)  0 (0) | 6 (85.7)  1 (14.3) | 22 (91.7)  2 (8.3) | 5 (83.3)  1 (16.7) | 0.49 (NS) |
| **HPV genotype**  Genotype 16  Other genotypes | 72 (90) 8 (10) | 28 (90.6)  3 (9.4) | 7 (100)  0 (0) | 3 (100)  0 (0) | 5 (71.4)  2 (28.6) | 24 (96)  1 (4) | 5 (71.4)  2 (28.6) | 0.19 (NS) |
| **Tumor differentiation**^d^  Well/Moderate  Poor | 32 (56.1)  25 (43.9) | 14 (51.9) 12 (48.1) | 0 (0)  3 (100) | 2 (100)  0 (0) | 1 (33.3)  2 (66.7) | 12 (75)  4 (25) | 3 (42.9)  4 (57.1) | 0.11 (NS) |
| **Mitotic index^e^**  Low/moderate  High | 8 (20)  32 (80) | 5 (22.7) 16 (77.3) | 0 (0)  0 (0) | 0 (0)  1 (100) | 0 (0)  1 (100) | 3 (25)  9 (75) | 0 (0)  5 (100) | NA |
| **Perineural invasion ^f^**  Yes  No | 12 (28.6)  30 (71.4) | 6 (37.5) 9 (62.5) | 0 (0)  4 (100) | 1 (33.3)  2 (66.7) | 1 (33.3)  2 (66.7) | 4 (26.7)  11 (73.3) | 0 (0)  2 (100) | 0.63 (NS) |
| **Lymphovascular invasion ^g^**  Yes  No | 17 (37)  29 (63) | 3 (20) 11 (80) | 3 (50)  3 (50) | 0 (0)  1 (100) | 2 (33.3)  4 (66.7) | 8 (50)  8 (50) | 1 (33.3)  2 (66.7) | 0.59 (NS) |
| **Initial therapy**^a^  Surgery with or without induction CT Exclusive (chemo) RT  Surgery followed by (chemo) radiation None | 8 (10.1)  15 (19)  54 (68.4)  2 (2.5) | 3 (9.7)  4 (12.9)  21 (70.9) 2 (6.5) | 1 (14.3)  0 (0)  6 (85.7)  0 (0) | 0 (0)  0 (0)  3 (100)  0 (0) | 1 (14.3)  2 (28.6)  4 (57.1)  0 (0) | 3 (12)  7 (28)  15 (60)  0 (0) | 0 (0)  2 (28.6)  5 (71.4)  0 (0) | 0.83 (NS) |
| **HPV copy number**  Low (<9)  High (≥9) | 36 (45)  44 (55) | 12 (40.5)  19 (59.5) | 5 (71.4)  2 (28.6) | 1 (33.3)  2 (66.7) | 3 (42.9)  4 (57.1) | 9 (36)  16 (64) | 6 (85.7)  1 (14.3) | 0.15 (NS) |

^a^ Data available for 79 patients; ^b^ Data available for 76 patients; ^c^ Data available for 75 patients; ^d^ Data available for 57 patients, ^e^ Data available for 40; ^f^ Data available for 42; ^g^ Data available for 46; ^h^ Chi-Square test *p* values for comparison of the EPI vs 2J-COL vs 2J-NL vs MJ-CL vs MJ-SC groups for each parameter; Tumor stage UICC 8e classification CT: chemotherapy; RT: radiotherapy
