# Supplementary material for: Human papilloma virus integration sites and genomic signatures in head and neck squamous cell carcinoma
Source: Mol Oncol. 2022 May 10;16(16):3001–16. doi: 10.1002/1878-0261.13219 (PMC9394244; doi:10.1002/1878-0261.13219)
Supplement: Supplementary file 12 — Table S5. HPV integration sites in human chromosomes of 49 HNSCC patients. [file MOL2-16-3001-s005.docx]

**Supplementary table 5:** HPV integration sites in human chromosomes of 49 HNSCC patients.

| **Patient ID** | **HPV type** | **HPV genomic signature** | **Chromosomal region** | **Genomic position (GRCh37)** | **Genomic position (GRCh38)** | **Intragenic** | **Intron/exon** | **5' genes** | **3'genes** | **Number of patients** |
| --- | --- | --- | --- | --- | --- | --- | --- | --- | --- | --- |
| R14 | HPV16 | MJ-SC | chr11q12.1 | 55 938 754 | 56 171 278 |  |  | *OR8K5* | *OR5J2* | 1 |
| R14 | HPV16 | MJ-SC | chr16p11.1 | 35 239 036 | 36 004 665 |  |  | *FLJ26245* | *C* | 1 |
| R14 | HPV16 | MJ-SC | chr16q23.1 | 77 013 478 | 76 979 581 |  |  | *mir4719* | *MON1B* | 1 |
| R14 | HPV16 | MJ-SC | chr2q37.1 | 234 321 431 | 233 412 785 | *DGKD* | intron |  |  | 1 |
| R14 | HPV16 | MJ-SC | chr4p12 | 47 968 644 | 47 966 627 | *CNGA1* | intron |  |  | 1 |
| R14 | HPV16 | MJ-SC | chr5p15.33 | 1 614 886 | 1 614 771 | *LOC728613* | intron |  |  | 1 |
| R14 | HPV16 | MJ-SC | chr6q11.1 | 61 902 807 | 61 334 393 |  |  | *C* | *KHDRBS2* | 1 |
| R290 | HPV16 | 2J | chr8p11.21 | 42 148 700 | 42 291 182 | *IKBKB* | intron |  |  | 1 |
| R294 | HPV16 | MJ-SC | chr20p12.1 | 14 681 174 | 14 700 528 | *MACROD2* | intron |  |  | 2 |
| R294 | HPV16 | MJ-SC | chr20p12.1 | 14 984 772 | 15 004 126 | *MACROD2* | intron |  |  | 2 |
| R294 | HPV16 | MJ-SC | chr20p12.1 | 14 989 643 | 15 008 997 | *MACROD2* | intron |  |  | 2 |
| R294 | HPV16 | MJ-SC | chr20p12.1 | 15 016 801 | 15 036 155 | *MACROD2* | intron |  |  | 2 |
| R294 | HPV16 | MJ-SC | chr6q14.1 | 79 206 499 | 78 496 782 |  |  | *IMPG1* | *IRAK1BP1* | 1 |
| R294 | HPV16 | MJ-SC | chr6q14.1 | 79 216 361 | 78 506 644 |  |  | *IMPG1* | *IRAK1BP1* | 1 |
| R294 | HPV16 | MJ-SC | chr6q14.1 | 79 225 358 | 78 515 641 |  |  | *IMPG1* | *IRAK1BP1* | 1 |
| R294 | HPV16 | MJ-SC | chr6q14.1 | 79 226 779 | 78 517 062 |  |  | *IMPG1* | *IRAK1BP1* | 1 |
| R295 | HPV35 | MJ-SC | chr17q25.3 | 77 801 666 | 79 827 867 |  |  | *LINC01977* | *CBX4* | 1 |
| R295 | HPV35 | MJ-SC | chr17q25.3 | 77 829 165 | 79 855 366 |  |  | *CBX4* | *TBC1D16* | 1 |
| R295 | HPV35 | MJ-SC | chr20q13.2 | 52 508 068 | 53 891 529 |  |  | *ZNF217* | *BCAS1* | 1 |
| R295 | HPV35 | MJ-SC | chr22q13.31 | 44 185 604 | 43 789 724 | *EFCAB6* | intron |  |  | 1 |
| R295 | HPV35 | MJ-SC | chr22q13.31 | 44 312 968 | 43 917 088 |  |  | *PNPLA5* | *PNPLA3* | 1 |
| R295 | HPV35 | MJ-SC | chr22q13.31 | 44 327 502 | 43 931 622 | *PNPLA3* | intron |  |  | 1 |
| R295 | HPV35 | MJ-SC | chr22q13.31 | 44 330 557 | 43 934 677 | *PNPLA3* | intron |  |  | 1 |
| R295 | HPV35 | MJ-SC | chr3p12.1 | 86 003 214 | 85 954 064 | *CADM2* | intron |  |  | 1 |
| R295 | HPV35 | MJ-SC | chr3p12.1 | 86 017 227 | 85 968 077 | *CADM2* | intron |  |  | 1 |
| R295 | HPV35 | MJ-SC | chr3p12.1 | 86 017 230 | 85 968 080 | *CADM2* | intron |  |  | 1 |
| R295 | HPV35 | MJ-SC | chr3p12.1 | 86 104 126 | 86 054 976 | *CADM2* | intron |  |  | 1 |
| R295 | HPV35 | MJ-SC | chr3p12.1 | 86 128 166 | 86 079 016 |  |  | *CADM2* | *C* | 1 |
| R295 | HPV35 | MJ-SC | chr9p24.1 | 5 424 212 | 5 424 212 | *PLGRKT* | intron |  |  | 4 |
| R295 | HPV35 | MJ-SC | chr9p24.1 | 5 430 785 | 5 430 785 | *PLGRKT* | intron |  |  | 4 |
| R295 | HPV35 | MJ-SC | chr9p24.1 | 5 430 809 | 5 430 809 | *PLGRKT* | intron |  |  | 4 |
| R295 | HPV35 | MJ-SC | chr9p24.1 | 5 431 105 | 5 431 105 | *PLGRKT* | intron |  |  | 4 |
| R295 | HPV35 | MJ-SC | chr9p24.1 | 5 432 251 | 5 432 251 | *PLGRKT* | intron |  |  | 4 |
| R295 | HPV35 | MJ-SC | chr9p24.1 | 5 436 954 | 5 436 954 | *PLGRKT* | intron |  |  | 4 |
| R295 | HPV35 | MJ-SC | chr9p24.1 | 5 437 673 | 5 437 673 | *PLGRKT* | intron |  |  | 4 |
| R295 | HPV35 | MJ-SC | chr9p24.1 | 5 449 830 | 5 449 830 | *CD274* | 673pb in 5’ |  |  | 4 |
| R295 | HPV35 | MJ-SC | chr9p24.1 | 5 453 753 | 5 453 753 | *CD274* | intron |  |  | 4 |
| R295 | HPV35 | MJ-SC | chr9p24.1 | 5 455 384 | 5 455 384 | *CD274* | intron |  |  | 4 |
| R295 | HPV35 | MJ-SC | chr9p24.1 | 5 455 438 | 5 455 438 | *CD274* | intron |  |  | 4 |
| R295 | HPV35 | MJ-SC | chr9p24.1 | 5 458 940 | 5 458 940 | *CD274* | intron |  |  | 4 |
| R295 | HPV35 | MJ-SC | chr9p24.1 | 5 460 047 | 5 460 047 | *CD274* | intron |  |  | 4 |
| R295 | HPV35 | MJ-SC | chr9p24.1 | 5 460 484 | 5 460 484 | *CD274* | intron |  |  | 4 |
| R295 | HPV35 | MJ-SC | chr9p24.1 | 5 463 224 | 5 463 224 | *CD274* | intron |  |  | 4 |
| R295 | HPV35 | MJ-SC | chr9p24.1 | 5 466 801 | 5 466 801 | *CD274* | exon |  |  | 4 |
| R295 | HPV35 | MJ-SC | chr9p24.1 | 5 469 806 | 5 469 806 | *CD274* | exon |  |  | 4 |
| R295 | HPV35 | MJ-SC | chr9p24.1 | 5 469 813 | 5 469 813 | *CD274* | exon |  |  | 4 |
| R299 | HPV33 | 2J | chr9p24.1 | 5 436 902 | 5 436 902 | *PLGRKT* | intron |  |  | 4 |
| R303 | HPV16 | MJ-SC | chr2p11.1 | 92 322 370 | 92 134 344 |  |  | *C* | *C* | 1 |
| R303 | HPV16 | MJ-SC | chr20q11.23 | 36 530 585 | 37 902 183 |  |  | *CTNNBL1* | *VSTM2L* | 1 |
| R303 | HPV16 | MJ-SC | chr5q31.1 | 134 598 462 | 135 262 772 | *LOC100996485, C5ORF66* | introns |  |  | 1 |
| R303 | HPV16 | MJ-SC | chr8q24.3 | 142 462 280 | 141 452 180 | *MROH5* | intron |  |  | 1 |
| R303 | HPV16 | MJ-SC | chr8q24.3 | 142 462 281 | 141 452 181 | *MROH5* | intron |  |  | 1 |
| R306 | HPV35 | 2J | chr7p12.3 | 48 516 705 | 48 477 108 | *ABCA13* | intron |  |  | 1 |
| R310 | HPV16 | 2J | chrYq11.21 | 15 026 693 | 12 914 781 | *DDX3Y* | intron |  |  | 1 |
| R313 | HPV16 | MJ-SC | chr16p13.12 | 13 412 263 | 13 318 406 |  |  | *SHISA9* | *ERCC4* | 1 |
| R313 | HPV16 | MJ-SC | chr20p12.2 | 11 345 697 | 11 365 049 |  |  | *LOC339593* | *BTBD3* | 1 |
| R313 | HPV16 | MJ-SC | chr5p15.32 | 5 983 735 | 5 983 622 |  |  | *KIAA0947* | *MED10* | 1 |
| R313 | HPV16 | MJ-SC | chr6q21 | 107 311 677 | 106 990 477 |  |  | *LOC100422737* | *C6orf203* | 1 |
| R313 | HPV16 | MJ-SC | chr6q21 | 107 316 509 | 106 995 305 |  |  | *LOC100422737* | *C6orf203* | 1 |
| R313 | HPV16 | MJ-SC | chr6q21 | 107 316 943 | 106 995 739 |  |  | *LOC100422737* | *C6orf203* | 1 |
| R313 | HPV16 | MJ-SC | chr8p11.23 | 36 931 551 | 37 074 033 |  |  | *KCNU1* | *X* | 1 |
| R313 | HPV16 | MJ-SC | chr8p11.23 | 36 931 916 | 37 074 398 |  |  | *KCNU1* | *X* | 1 |
| R314 | HPV16 | MJ-SC | chr1p21.1 | 104 587 958 | 104 045 336 |  |  | *AMY1A* | *NTNG1* | 1 |
| R314 | HPV16 | MJ-SC | chr1p21.1 | 104 611 154 | 104 068 532 |  |  | *AMY1A* | *NTNG1* | 1 |
| R314 | HPV16 | MJ-SC | chr1p21.1 | 104 655 782 | 104 113 160 |  |  | *AMY1A* | *NTNG1* | 1 |
| R314 | HPV16 | MJ-SC | chr1p21.1 | 104 660 077 | 104 117 455 |  |  | *AMY1A* | *NTNG1* | 1 |
| R314 | HPV16 | MJ-SC | chr1p21.1 | 104 735 905 | 104 193 283 |  |  | *AMY1A* | *NTNG1* | 1 |
| R314 | HPV16 | MJ-SC | chr1p21.1 | 178 066 867 | 178 097 732 | *RASAL2* | intron |  |  | 1 |
| R314 | HPV16 | MJ-SC | chr2q31.1 | 177 773 847 | 176 909 119 |  |  | *LOC375295* | *HNRNPA3* | 1 |
| R314 | HPV16 | MJ-SC | chr2q31.1 | 178 203 608 | 177 338 880 | *NFE2L2* | intron |  |  | 1 |
| R314 | HPV16 | MJ-SC | chr5q35.3 | 180 127 324 | 180 700 324 |  |  | *FLT4* | *MGAT1* | 1 |
| R315 | HPV56 | MJ-SC | chr13q22.1 | 73 622 374 | 73 048 236 |  |  | *PIBF1* | *KLF5* | 2 |
| R315 | HPV56 | MJ-SC | chr13q22.1 | 73 679 131 | 73 104 994 |  |  | *PIBF1* | *KLF5* | 2 |
| R315 | HPV56 | MJ-SC | chr19p13.2 | 10 573 117 | 10 462 441 | *PDE4A* | intron |  |  | 1 |
| R315 | HPV56 | MJ-SC | chr19p13.2 | 10 652 600 | 10 541 924 |  |  | *S1PR5* | *ATG4D* | 1 |
| R318 | HPV16 | 2J-NL | chr16p13.13 | 10 920 860 | 10 827 003 |  |  | *TVP23A* | *CIITA* | 1 |
| R318 | HPV16 | 2J-NL | chr16p13.13 | 10 950 834 | 10 856 977 |  |  | *TVP23A* | *CIITA* | 1 |
| R320 | HPV16 | MJ-SC | chr10q11.21 | 42 529 386 | 41 710 823 |  |  | *C* | *LOC441666* | 1 |
| R320 | HPV16 | MJ-SC | chr11q24.1 | 121 953 183 | 122 082 475 | *MIR100HG* | intron |  |  | 1 |
| R320 | HPV16 | MJ-SC | chr11q24.1 | 122 022 535 | 122 151 827 | *MIR100HG* | intron |  |  | 1 |
| R320 | HPV16 | MJ-SC | chr11q24.1 | 122 036 022 | 122 165 314 | *MIR100HG* | intron |  |  | 1 |
| R320 | HPV16 | MJ-SC | chr11q24.1 | 122 037 260 | 122 166 552 | *MIR100HG* | intron |  |  | 1 |
| R320 | HPV16 | MJ-SC | chr11q24.1 | 122 037 708 | 122 167 000 | *MIR100HG* | intron |  |  | 1 |
| R320 | HPV16 | MJ-SC | chr14q12 | 27 558 170 | 27 088 964 |  |  | *NOVA1* | *MIR4307* | 1 |
| R320 | HPV16 | MJ-SC | chr14q12 | 27 558 179 | 27 088 973 |  |  | *NOVA1* | *MIR4307* | 1 |
| R320 | HPV16 | MJ-SC | chr2p15 | 63 348 433 | 63 121 298 | *WDPCP* | exon |  |  | 1 |
| R320 | HPV16 | MJ-SC | chrXp11.4 | 39 708 365 | 39 849 111 |  |  | *MID1* | *BCOR* | 1 |
| R320 | HPV16 | MJ-SC | chrXp11.4 | 39 844 420 | 39 985 166 |  |  | *MID1* | *BCOR* | 1 |
| R320 | HPV16 | MJ-SC | chrXp11.4 | 39 844 432 | 39 985 178 |  |  | *MID1* | *BCOR* | 1 |
| R320 | HPV16 | MJ-SC | chrYq11.223 | 23 992 656 | 21 846 509 |  |  | *RBMY1B* | *RBMY1B* | 1 |
| R320 | HPV16 | MJ-SC | chrYq11.223 | 24 023 805 | 21 877 658 |  |  | *RBMY1B* | *RBMY1B* | 1 |
| R321 | HPV16 | 2J | chr1q41 | 223 410 090 | 223 236 748 | *SUSD4* | intron |  |  | 1 |
| R322 | HPV16 | MJ-SC | chr12p13.33 | 1 806 455 | 1 697 289 | *ADOPOR2* | intron |  |  | 1 |
| R322 | HPV16 | MJ-SC | chr12q24.23 | 119 623 585 | 119 185 780 | *HSPB8* | intron |  |  | 1 |
| R322 | HPV16 | MJ-SC | chr6p12.3 | 47 208 966 | 47 241 230 | TNFRSF21 | intron |  |  | 1 |
| R322 | HPV16 | MJ-SC | chr6p12.3 | 47 209 004 | 47 241 268 | TNFRSF21 | intron |  |  | 1 |
| R548 | HPV16 | 2J-COL | chr17q25.3 | 80 795 386 | 82 837 510 | *TBCD, ZNF750* | introns |  |  | 1 |
| R548 | HPV16 | 2J-COL | chr17q25.3 | 80 795 476 | 82 837 600 | *TBCD, ZNF750* | introns |  |  | 1 |
| R550 | HPV16 | MJ-CL | chr17q12 | 35 202 656 | 36 845 391 |  |  | *MRM1* | *LHX1-DT* | 1 |
| R550 | HPV16 | MJ-CL | chr17q12 | 35 265 144 | 36 907 859 | *LHX1-DT* | intron |  |  | 1 |
| R550 | HPV16 | MJ-CL | chr17q21.31 | 42 691 696 | 44 614 328 |  |  | *FZD2* | *C17orf104* | 1 |
| R550 | HPV16 | MJ-CL | chr17q21.31 | 42 708 985 | 44 631 617 |  |  | *FZD2* | *C17orf104* | 1 |
| R550 | HPV16 | MJ-CL | chr17q21.31 | 42 720 459 | 44 643 091 |  |  | *FZD2* | *C17orf104* | 1 |
| R550 | HPV16 | MJ-CL | chr17q21.31 | 42 721 490 | 44 644 122 |  |  | *FZD2* | *C17orf104* | 1 |
| R550 | HPV16 | MJ-CL | chr17q21.31 | 42 745 558 | 44 668 190 | *C17ORF104* | exon |  |  | 1 |
| R550 | HPV16 | MJ-CL | chr17q21.31 | 42 799 983 | 44 722 615 | *DBF4B* | intron |  |  | 1 |
| R592 | HPV16 | MJ-SC | chr12q13.13 | 53 994 655 | 53 600 871 | *ATF7* | intron |  |  | 1 |
| R592 | HPV16 | MJ-SC | chr17q24.3 | 70 619 437 | 72 623 298 | *LINC00511* | intron |  |  | 1 |
| R592 | HPV16 | MJ-SC | chr17q24.3 | 70 619 523 | 72 623 384 | *LINC00511* | intron |  |  | 1 |
| R592 | HPV16 | MJ-SC | chr2p11.1 | 92 316 071 | 92 128 045 |  |  | *ACTR3BP2* | *C* | 1 |
| R592 | HPV16 | MJ-SC | chr2q24.23 | 162 179 283 | 161 322 772 | *PSMD14* | intron |  |  | 1 |
| R593 | HPV16 | MJ-SC | chr13q22.1 | 73 932 581 | 73 358 444 |  |  | *KLF5* | *KLF12* | 2 |
| R593 | HPV16 | MJ-SC | chr2p13.2 | 73 221 203 | 72 994 074 | *SFXN5* | intron |  |  | 1 |
| R595 | HPV16 | MJ-SC | chr13q21.31 | 63 770 411 | 63 196 278 | *LIN00376* | intron |  |  | 1 |
| R595 | HPV16 | MJ-SC | chr14q21.3 | 49 594 378 | 49 127 660 |  |  | *LINC00648* | *NEMF* | 1 |
| R595 | HPV16 | MJ-SC | chr15q13.1 | 28 125 822 | 27 880 676 | *OCA2* | intron |  |  | 1 |
| R595 | HPV16 | MJ-SC | chr17q21.2 | 38 642 185 | 40 485 933 | *TNS4* | intron |  |  | 1 |
| R595 | HPV16 | MJ-SC | chr17q21.2 | 38 642 208 | 40 485 956 | *TNS4* | intron |  |  | 1 |
| R595 | HPV16 | MJ-SC | chr17q21.2 | 38 645 356 | 40 489 104 | *TNS4* | intron |  |  | 1 |
| R595 | HPV16 | MJ-SC | chr18q12.1 | 26 630 745 | 29 050 781 |  |  | *CDH2* | *MIR302F* | 1 |
| R595 | HPV16 | MJ-SC | chr4q13.1 | 61 152 905 | 60 287 187 |  |  | *REST* | *LPHN3* | 1 |
| R595 | HPV16 | MJ-SC | chr5p14.3 | 18 890 676 | 18 890 567 |  |  | *BASP1* | *CDH18* | 1 |
| R595 | HPV16 | MJ-SC | chr6q22.31 | 120 615 682 | 120 294 536 |  |  | *LOC285762* | *c6orf170* | 1 |
| R595 | HPV16 | MJ-SC | chr6q22.31 | 121 572 073 | 121 250 927 | *c6orf170* | intron |  |  | 1 |
| R595 | HPV16 | MJ-SC | chr7q32.3 | 130 482 876 | 130 798 117 |  |  | *KLF14* | *LOC646329* | 1 |
| R595 | HPV16 | MJ-SC | chr9q21.13 | 77 938 431 | 75 323 515 |  |  | *OSTF1* | *MIR548H3* | 1 |
| R596 | HPV16 | MJ-SC | chr14q23.2 | 62 362 741 | 61 896 023 | *SYT16* | intron |  |  | 1 |
| R596 | HPV16 | MJ-SC | chr2p12 | 81 793 265 | 81 566 141 |  |  | *CTNNA2* | *SUCLG1* | 1 |
| R607 | HPV16 | 2J-NL | chr11p15.4 | 7 692 723 | 7 671 492 | *CYB5R2* | intron |  |  | 1 |
| R607 | HPV16 | 2J-NL | chr11p15.4 | 7 692 802 | 7 671 571 | *CYB5R2* | intron |  |  | 1 |
| R608 | HPV16 | 2J-COL | chr14q23.2 | 69 259 955 | 68 793 238 | *ZFP36L1* | exon |  |  | 1 |
| R608 | HPV16 | 2J-COL | chr14q23.2 | 69 261 073 | 68 794 356 | *ZFP36L1* | intron |  |  | 1 |
| R613 | HPV16 | MJ-CL | chr9p24.1 | 5 429 919 | 5 429 919 | *PLGRKT* | intron |  |  | 4 |
| R613 | HPV16 | MJ-CL | chr9p24.1 | 5 451 420 | 5 451 420 | *CD274* | intron |  |  | 4 |
| R613 | HPV16 | MJ-CL | chr9p24.1 | 5 453 432 | 5 453 432 | *CD274* | intron |  |  | 4 |
| R613 | HPV16 | MJ-CL | chr9p24.1 | 5 453 433 | 5 453 433 | *CD274* | intron |  |  | 4 |
| R613 | HPV16 | MJ-CL | chr9p24.1 | 5 467 946 | 5 467 946 | *CD274* | exon |  |  | 4 |
| R613 | HPV16 | MJ-CL | chr9p24.1 | 5 469 019 | 5 469 019 | *CD274* | exon |  |  | 4 |
| R617 | HPV16 | 2J-COL | chr14q31.3 | 86 425 338 | 85 958 994 | *LINC02328* | intron |  |  | 1 |
| R617 | HPV16 | 2J-COL | chr14q31.3 | 86 426 548 | 85 960 204 | *LINC02328* | intron |  |  | 1 |
| R619 | HPV16 | MJ-SC | chr1q22 | 155 681 140 | 155 711 349 | *DAP3* | intron |  |  | 1 |
| R619 | HPV16 | MJ-SC | chr12q24.32 | 126 573 545 | 126 088 999 |  |  | *LINC00939* | *LOC100128554* | 1 |
| R619 | HPV16 | MJ-SC | chr12q24.32 | 126 574 356 | 126 089 810 |  |  | *LINC00939* | *LOC100128554* | 1 |
| R619 | HPV16 | MJ-SC | chr12q24.32 | 126 583 788 | 126 099 242 |  |  | *LINC00939* | *LOC100128554* | 1 |
| R619 | HPV16 | MJ-SC | chr12q24.32 | 126 583 791 | 126 099 245 |  |  | *LINC00939* | *LOC100128554* | 1 |
| R619 | HPV16 | MJ-SC | chr12q24.32 | 126 592 041 | 126 107 495 |  |  | *LINC00939* | *LOC100128554* | 1 |
| R619 | HPV16 | MJ-SC | chr12q24.32 | 126 618 932 | 126 134 386 |  |  | *LINC00939* | *LOC100128554* | 1 |
| R619 | HPV16 | MJ-SC | chr12q24.32 | 126 639 522 | 126 154 976 |  |  | *LINC00939* | *LOC100128554* | 1 |
| R619 | HPV16 | MJ-SC | chr12q24.32 | 126 666 190 | 126 181 644 |  |  | *LINC00939* | *LOC100128554* | 1 |
| R619 | HPV16 | MJ-SC | chr13q34 | 110 392 007 | 109 739 660 |  |  | *MYO16* | *IRS2* | 1 |
| R619 | HPV16 | MJ-SC | chr2q31.1 | 176 048 176 | 175 183 448 |  |  | *ATP5G3* | *X* | 1 |
| R619 | HPV16 | MJ-SC | chr4q32.1 | 160 139 727 | 159 218 575 | *RAPGEF2* | intron |  |  | 1 |
| R619 | HPV16 | MJ-SC | chr6q14.1 | 81 286 474 | 80 576 757 |  |  | *BCKDHB* | *FAM46A* | 1 |
| R619 | HPV16 | MJ-SC | chr7p22.1 | 5 121 225 | 5 081 594 |  |  | *RBAK* | *ZNF890P* | 1 |
| R619 | HPV16 | MJ-SC | chr8q22.1 | 97 266 634 | 96 254 406 | *MTERFD1* | intron |  |  | 1 |
| R619 | HPV16 | MJ-SC | chr8q22.1 | 97 267 432 | 96 255 204 | *MTERFD1* | intron |  |  | 1 |
| R619 | HPV16 | MJ-SC | chr8q22.2 | 101 454 216 | 100 441 988 |  |  | *MIR4471* | *ANKRD46* | 1 |
| R619 | HPV16 | MJ-SC | chr8q22.2 | 101 470 939 | 100 458 711 |  |  | *MIR4471* | *ANKRD46* | 1 |
| R619 | HPV16 | MJ-SC | chr8q22.2 | 101 475 839 | 100 463 611 |  |  |  |  | 1 |
| R619 | HPV16 | MJ-SC | chr8q22.2 | 101 532 221 | 100 519 993 | *ANKRD46* | intron |  |  | 1 |
| R619 | HPV16 | MJ-SC | chr8q24.21 | 128 874 012 | 127 861 766 | *PVT1* | intron |  |  | 3 |
| R619 | HPV16 | MJ-SC | chr8q24.21 | 128 874 013 | 127 861 767 | *PVT1* | intron |  |  | 3 |
| R619 | HPV16 | MJ-SC | chr8q24.21 | 128 874 014 | 127 861 768 | *PVT1* | intron |  |  | 3 |
| R619 | HPV16 | MJ-SC | chr8q24.21 | 128 874 015 | 127 861 769 | *PVT1* | intron |  |  | 3 |
| R619 | HPV16 | MJ-SC | chr8q24.21 | 128 912 729 | 127 900 483 | *PVT1* | intron |  |  | 3 |
| R619 | HPV16 | MJ-SC | chr8q24.21 | 128 914 816 | 127 902 570 | *PVT1* | intron |  |  | 3 |
| R619 | HPV16 | MJ-SC | chr8q24.21 | 128 920 785 | 127 908 539 | *PVT1* | intron |  |  | 3 |
| R620 | HPV16 | MJ-CL | chr9q22.33 | 100 596 966 | 97 834 684 | *PTCSC2* |  |  |  | 1 |
| R620 | HPV16 | MJ-CL | chr9q22.33 | 100 597 050 | 97 834 768 | *PTCSC2* |  |  |  | 1 |
| R620 | HPV16 | MJ-CL | chr9q22.33 | 100 639 515 | 97 877 233 |  |  | *XPA* | *FOXE1* | 1 |
| R621 | HPV33 | MJ-CL | chr3p21.31 | 49 125 202 | 49 087 769 | *QRICH1* | intron |  |  | 1 |
| R621 | HPV33 | MJ-CL | chr3p21.31 | 49 125 904 | 49 088 471 | *QRICH1* | intron |  |  | 1 |
| R621 | HPV33 | MJ-CL | chr3p21.31 | 49 282 228 | 49 244 795 | *CCDC36* | intron |  |  | 1 |
| R621 | HPV33 | MJ-CL | chr3p21.31 | 49 291 248 | 49 253 815 | *CCDC36* | intron |  |  | 1 |
| R623 | HPV16 | MJ-SC | chr12q13.13 | 54 322 659 | 53 928 875 |  |  | *CALCOCO1* | *HOXC13-AS* | 1 |
| R623 | HPV16 | MJ-SC | chr12q13.13 | 54 322 676 | 53 928 892 |  |  | *CALCOCO1* | *HOXC13-AS* | 1 |
| R623 | HPV16 | MJ-SC | chr12q22 | 93 648 690 | 93 254 914 | *LOC643339* | intron |  |  | 1 |
| R623 | HPV16 | MJ-SC | chr12q22 | 93 648 916 | 93 255 140 | *LOC643339* | intron |  |  | 1 |
| R623 | HPV16 | MJ-SC | chr4q34.3 | 182 275 459 | 181 354 306 |  |  | *LINC00290* | *X* | 1 |
| R623 | HPV16 | MJ-SC | chr4q34.3 | 182 280 941 | 181 359 788 |  |  | *LINC00290* | *X* | 1 |
| R623 | HPV16 | MJ-SC | chr7q21.11 | 78 404 156 | 78 774 840 | *MAGI2* | intron |  |  | 1 |
| R623 | HPV16 | MJ-SC | chr7q21.11 | 78 406 517 | 78 777 201 | *MAGI2* | intron |  |  | 1 |
| R624 | HPV16 | MJ-SC | chr1p21.2 | 100 998 341 | 100 532 785 |  |  | *CDC14A* | *GPR88* | 1 |
| R624 | HPV16 | MJ-SC | chr16p12.1 | 25 429 078 | 25 417 757 |  |  | *ZKSCAN2* | *HST3ST4* | 1 |
| R624 | HPV16 | MJ-SC | chr16p12.1 | 25 436 061 | 25 424 740 |  |  | *ZKSCAN2* | *HST3ST4* | 1 |
| R624 | HPV16 | MJ-SC | chr16p12.1 | 25 440 083 | 25 428 762 |  |  | *ZKSCAN2* | *HST3ST4* | 1 |
| R624 | HPV16 | MJ-SC | chr7q31.1 | 110 823 730 | 111 183 674 | *IMMP2L* | intron |  |  | 1 |
| R624 | HPV16 | MJ-SC | chrXp11.22 | 53 029 707 | 53 000 528 |  |  | *FAM156A* | *GPR173* | 1 |
| R624 | HPV16 | MJ-SC | chrXp11.22 | 53 051 374 | 53 022 192 |  |  | *FAM156A* | *GPR173* | 1 |
| R626 | HPV16 | 2J-COL | chr1q32.2 | 209 560 836 | 209 387 491 |  |  | *X* | *MIR205* | 1 |
| R626 | HPV16 | 2J-COL | chr1q32.2 | 209 637 193 | 209 463 848 |  |  | *X* | *MIR205* | 1 |
| R628 | HPV16 | MJ-SC | chrXq21.31 | 87 953 695 | 88 698 694 |  |  | *KLHL4* | *CPXCR1* | 1 |
| R628 | HPV16 | MJ-SC | chrYq11.221 | 19 054 004 | 16 942 124 |  |  | *NLGN4Y* | *FAM41AY2* | 1 |
| R629 | HPV16 | MJ-SC | chr11q13.4 | 70 535 145 | 70 689 040 | *SHANK2* | intron |  |  | 1 |
| R629 | HPV16 | MJ-SC | chr11q13.4 | 70 541 772 | 70 695 667 | *SHANK2* | intron |  |  | 1 |
| R629 | HPV16 | MJ-SC | chr11q13.4 | 70 541 774 | 70 695 669 | *SHANK2* | intron |  |  | 1 |
| R629 | HPV16 | MJ-SC | chr11q13.4 | 70 543 535 | 70 697 430 | *SHANK2* | intron |  |  | 1 |
| R629 | HPV16 | MJ-SC | chr11q13.4 | 70 553 555 | 70 707 450 | *SHANK2* | intron |  |  | 1 |
| R629 | HPV16 | MJ-SC | chr15q11.2 | 24 084 524 | 23 839 377 |  |  | *NDN* | *PWRN1* | 1 |
| R629 | HPV16 | MJ-SC | chr19p13.3 | 3 727 019 | 3 727 021 | *TJP3* | intron |  |  | 1 |
| R629 | HPV16 | MJ-SC | chr8q22.2 | 100 317 138 | 99 304 910 | *VPS13B* | intron |  |  | 1 |
| R629 | HPV16 | MJ-SC | chr8q22.2 | 100 326 978 | 99 314 750 | *VPS13B* | intron |  |  | 1 |
| R629 | HPV16 | MJ-SC | chr8q22.2 | 100 351 779 | 99 339 551 | *VPS13B* | intron |  |  | 1 |
| R629 | HPV16 | MJ-SC | chr8q22.2 | 100 379 960 | 99 367 732 | *VPS13B* | intron |  |  | 1 |
| R629 | HPV16 | MJ-SC | chr9q21.11 | 71 778 481 | 69 163 565 | *TJP2* | intron |  |  | 1 |
| R631 | HPV16 | 2J-COL | chr22q11.22 | 22 315 529 | 21 961 157 | *TOP3B* | intron |  |  | 1 |
| R631 | HPV16 | 2J-COL | chr22q11.22 | 22 315 539 | 21 961 167 | *TOP3B* | intron |  |  | 1 |
| R632 | HPV16 | MJ-SC | chr16p12.2 | 23 335 811 | 23 324 490 | *SCNNB1* | intron |  |  | 1 |
| R632 | HPV16 | MJ-SC | chr20p12.1 | 15 343 031 | 15 362 385 | *MACROD2* | intron |  |  | 2 |
| R632 | HPV16 | 2J-COL | chr7p14.3 | 32 976 776 | 32 937 164 | *AVL9, RP9P* | introns |  |  | 1 |
| R633 | HPV26 | MJ-CL | chr9q31.2 | 110 328 572 | 107 566 291 |  |  | *KLF4* | *ACTL7B* | 1 |
| R633 | HPV26 | MJ-CL | chr9q31.2 | 110 905 032 | 108 142 752 |  |  | *KLF4* | *ACTL7B* | 1 |
| R633 | HPV26 | MJ-CL | chr9q31.2 | 110 924 180 | 108 161 900 |  |  | *KLF4* | *ACTL7B* | 1 |
| R635 | HPV16 | MJ-SC | chr10p15.1 | 4 797 717 | 4 755 525 |  |  | *KLF6* | *AKR1E2* | 1 |
| R635 | HPV16 | MJ-SC | chr4p16.3 | 1 789 728 | 1 788 001 |  |  | *TACC3* | *FGFR3* | 1 |
| R635 | HPV16 | MJ-SC | chr4p16.3 | 1 794 739 | 1 793 012 |  |  | *TACC3* | *FGFR3* | 1 |
| R636 | HPV16 | MJ-SC | chr22q12.3 | 34 043 495 | 33 647 509 | *LARGE* | intron |  |  | 1 |
| R636 | HPV16 | MJ-SC | chr22q12.3 | 36 082 618 | 35 686 571 |  |  | *APOL6* | *APOL5* | 1 |
| R636 | HPV16 | MJ-SC | chr22q12.3 | 36 103 708 | 35 707 661 |  |  | *APOL6* | *APOL5* | 1 |
| R646 | HPV16 | 2J-COL | chr14q21.2 | 46 278 043 | 45 808 840 |  |  | *MIS18BP1* | *LINC00871* | 1 |
| R646 | HPV16 | 2J-COL | chr14q21.2 | 46 278 101 | 45 808 898 |  |  | *MIS18BP1* | *LINC00871* | 1 |
| R647 | HPV16 | 2J | chr19q13.43 | 59 086 262 | 58 574 895 |  |  | *MZF1* | *MGC2752* | 1 |
| R648 | HPV16 | MJ-CL | chr2q37.3 | 242 006 420 | 241 067 004 | *SNED1* | intron |  |  | 1 |
| R648 | HPV16 | MJ-CL | chr9q31.1 | 107 538 345 | 104 776 064 |  |  | *NIPSNAP3A* | *ABCA1* | 1 |
| R648 | HPV16 | MJ-CL | chrXp22.12 | 19 584 287 | 19 566 169 | *SH3KBP1* | intron |  |  | 1 |
| R648 | HPV16 | MJ-CL | chrXp22.12 | 19 625 968 | 19 607 850 | *SH3KBP1* | intron |  |  | 1 |
| R648 | HPV16 | MJ-CL | chrXp22.12 | 19 625 983 | 19 607 865 | *SH3KBP1* | intron |  |  | 1 |
| R648 | HPV16 | MJ-CL | chrXp22.12 | 19 635 112 | 19 616 994 | *SH3KBP1* | intron |  |  | 1 |
| R648 | HPV16 | MJ-CL | chrXp22.12 | 19 635 356 | 19 617 238 | *SH3KBP1* | intron |  |  | 1 |
| R648 | HPV16 | MJ-CL | chrXp22.12 | 19 644 573 | 19 626 455 | *SH3KBP1* | intron |  |  | 1 |
| R648 | HPV16 | MJ-CL | chrXp22.12 | 19 681 081 | 19 662 963 | *SH3KBP1* | intron |  |  | 1 |
| R650 | HPV16 | MJ-SC | chr6q16.3 | 104 229 485 | 103 781 610 |  |  | *X* | *X* | 1 |
| R650 | HPV16 | MJ-SC | chr6q16.3 | 104 229 541 | 103 781 666 |  |  | *X* | *X* | 1 |
| R650 | HPV16 | MJ-SC | chr9p24.1 | 5 601 757 | 5 601 757 | *PDCD1LG2* | 30475 pb in 5’ |  |  | 4 |
| R650 | HPV16 | MJ-SC | chr9p24.1 | 5 603 718 | 5 603 718 | *PDCD1LG2* | 32436pb in 5’ |  |  | 4 |
| R653 | HPV16 | 2J | chr22q11.1 | 17 760 542 | 17 279 652 |  |  | *CECR1* | *CECR2* | 1 |
| R654 | HPV16 | MJ-CL | chr8q24.21 | 128 877 959 | 127 865 713 | *PVT1* | intron |  |  | 3 |
| R654 | HPV16 | MJ-CL | chr8q24.21 | 128 885 291 | 127 873 045 | *PVT1* | intron |  |  | 3 |
| R654 | HPV16 | MJ-CL | chr8q24.21 | 128 890 308 | 127 878 062 | *PVT1* | intron |  |  | 3 |
| R654 | HPV16 | MJ-CL | chr8q24.21 | 128 890 322 | 127 878 076 | *PVT1* | intron |  |  | 3 |
| R654 | HPV16 | MJ-CL | chr8q24.21 | 128 890 322 | 127 878 076 | *PVT1* | intron |  |  | 3 |
| R654 | HPV16 | MJ-CL | chr8q24.21 | 128 890 323 | 127 878 077 | *PVT1* | intron |  |  | 3 |
| R654 | HPV16 | MJ-CL | chr8q24.21 | 128 890 403 | 127 878 157 | *PVT1* | intron |  |  | 3 |
| R654 | HPV16 | MJ-CL | chr8q24.21 | 128 892 005 | 127 879 759 | *PVT1* | intron |  |  | 3 |
| R654 | HPV16 | MJ-CL | chr8q24.21 | 128 901 891 | 127 889 645 | *PVT1* | intron |  |  | 3 |
| R657 | HPV16 | MJ-SC | chr11p15.4 | 4 901 255 | 4 880 025 |  |  | *OR51S1* | *OR51T1* | 1 |
| R657 | HPV16 | MJ-SC | chr11p15.4 | 4 901 278 | 4 880 048 |  |  | *OR51S1* | *OR51T1* | 1 |
| R657 | HPV16 | MJ-SC | chr6q15 | 91 081 503 | 90 371 784 |  |  | *BACH2* | *MAP3K7* | 1 |
| R657 | HPV16 | MJ-SC | chr6q15 | 91 123 644 | 90 413 925 |  |  | *BACH2* | *MAP3K7* | 1 |
| R657 | HPV16 | MJ-SC | chr6q15 | 91 131 548 | 90 421 829 |  |  | *BACH2* | *MAP3K7* | 1 |
| R658 | HPV16 | 2J-NL | chr11p14.1 | 27 351 066 | 27 329 519 |  |  | *BBOX1* | *CCDC34* | 1 |
| R658 | HPV16 | 2J-NL | chr11p14.1 | 27 352 151 | 27 330 604 |  |  | *BBOX1* | *CCDC34* | 1 |
| R659 | HPV16 | 2J-COL | chr10q24.1 | 99 026 056 | 97 266 299 | *ARHGAP19* | intron |  |  | 1 |
| R659 | HPV16 | 2J-COL | chr10q24.1 | 99 026 075 | 97 266 318 | *ARHGAP19* | intron |  |  | 1 |
| R660 | HPV16 | MJ-SC | chr7q36.1 | 149 853 639 | 150 156 550 | *ACTRC3* | intron |  |  | 1 |
| R660 | HPV16 | MJ-SC | chr7q36.1 | 149 853 641 | 150 156 552 | *ACTRC3* | intron |  |  | 1 |
| R660 | HPV16 | MJ-SC | chr7q36.1 | 149 854 438 | 150 157 349 | *ACTRC3* | intron |  |  | 1 |
| R660 | HPV16 | MJ-SC | chr7q36.2 | 153 506 989 | 153 809 904 | *DPP6* | intron |  |  | 1 |
| R660 | HPV16 | MJ-SC | chr7q36.2 | 153 507 786 | 153 810 701 | *DPP6* |  |  |  | 1 |
| R660 | HPV16 | MJ-SC | chrXp11.3 | 43 637 236 | 43 777 989 | *MAOB* | Intron |  |  | 1 |
| R661 | HPV16 | MJ-SC | chr19p13.3 | 2 077 254 | 2 077 255 | *MOB3A* | intron |  |  | 1 |
| R661 | HPV16 | MJ-SC | chr8q24.21 | 128 136 896 | 127 124 651 |  |  | *PCAT* | *JX003871* | 1 |
| R661 | HPV16 | MJ-SC | chr8q24.21 | 128 151 831 | 127 139 586 |  |  | *PCAT* | *JX003871* | 1 |
| R661 | HPV16 | MJ-SC | chr8q24.21 | 128 781 750 | 127 769 504 |  |  | *MYC* | *PVT1* | 3 |
| R661 | HPV16 | MJ-SC | chr8q24.21 | 128 801 075 | 127 788 829 |  |  | *MYC* | *PVT1* | 3 |
| R661 | HPV16 | MJ-SC | chr8q24.21 | 128 806 354 | 127 794 108 |  |  | *MYC* | *PVT1* | 3 |
| R661 | HPV16 | MJ-SC | chr8q24.21 | 128 806 371 | 127 794 125 |  |  | *MYC* | *PVT1* | 3 |

**X :** no gene within 10^6^ pb
**C:** centromere
**HNSCC:** Head and Neck Squamous Cell Carcinoma

**Number of patients:** number of patients with HPV integration in the chromosomal region
