# Supplementary material for: Human papilloma virus integration sites and genomic signatures in head and neck squamous cell carcinoma
Source: Mol Oncol. 2022 May 10;16(16):3001–16. doi: 10.1002/1878-0261.13219 (PMC9394244; doi:10.1002/1878-0261.13219)
Supplement: Supplementary file 13 — Table S6. Associations between HPV genomic signatures and mRNA expression levels of immune‐related genes, EMT genes, cell proliferation genes and APOBEC genes. [file MOL2-16-3001-s004.docx]

**Supplementary Table 6.** Associations between HPV genomic signatures and mRNA expression levels of immune-related genes, EMT genes, cell proliferation genes and APOBEC genes.

^a^ : Kruskall Wallis test (EPI vs 2J vs MJ) (nonparametric test)

^b^ : One-way ANOVA (EPI vs 2J vs MJ) (parametric test)

N: normal tissues

EPI: episomal, 2J: two hybrid junctions, MJ: Multiple hybrid junctions
EMT: epitheliomesenchymal transformation

MDSC: Myeloid derived suppressive cells
